# IFSO Bariatric Endoscopy Committee Evidence-Based Review and Position Statement on Endoscopic Sleeve Gastroplasty for Obesity Management

**DOI:** 10.1007/s11695-024-07510-z

**Published:** 2024-11-01

**Authors:** Barham K. Abu Dayyeh, Christine Stier, Aayed Alqahtani, Reem Sharaiha, Mohit Bandhari, Silvana Perretta, Sigh Pichamol Jirapinyo, Gerhard Prager, Ricardo V. Cohen

**Affiliations:** 1 Mayo Clinic, Rochester, USA; 2 University Medical Centre Mannheim, Mannheim, Germany; 3 King Abdulaziz University, Jeddah, Saudi Arabia; 4 Cornell University, Ithaca, USA; 5 Sri Aurobindo Medical College and PG Institute, Indore, India; 6 IRCAD, Strasbourg, France; 7 Brigham and Women’s Hospital, Boston, USA; 8 Medical University of Vienna, Vienna, Austria; 9 The Center for Obesity and Diabetes, Oswaldo Cruz German Hospital, San Paolo, Brazil; 10 Gastroenterology and Advanced Endoscopy, Cedars-Sinai Health System, Los Angeles, USA

**Keywords:** Obesity, Endoscopic Sleeve Gastroplasty, Meta-analysis

## Abstract

**Background:**

Obesity is a significant global health issue. Metabolic and bariatric surgery (MBS) is the gold standard in the treatment of obesity due to its proven effectiveness and safety in the short and long term. However, MBS is not suitable for all patients. Some individuals are at high surgical risk or refuse surgical treatment, while others do not meet the criteria for MBS despite having obesity-related comorbidities. This gap has driven the development of endoscopic solutions like endoscopic sleeve gastroplasty (ESG), which offers a less invasive alternative that preserves organ function and reduces risks. A recent IFSO International Delphi consensus study highlighted that multidisciplinary experts agree on the utility of ESG for managing obesity in patients with class I and II obesity and for those with class III obesity who do not wish to pursue or qualify for MBS. This IFSO Bariatric Endoscopy Committee position statement aims to augment these consensus statements by providing a comprehensive systematic review of the evidence and delivering an evidence-based position on the value of ESG within the spectrum of obesity management.

**Methods:**

A comprehensive systematic review followed the Preferred Reporting Items for Systematic Reviews and Meta-analyses (PRISMA) and Cochrane guidelines.

**Results:**

*Systematic Review:* The systematic review included 44 articles encompassing 15,714 patients receiving ESG. The studies varied from large case series to cohort studies and a randomized controlled trial (RCT). The mean baseline BMI was 37.56 kg/m2. The review focused on weight loss outcomes and safety data.

*Meta-analysis:*

**Table:** 

Time point	Mean %EWL	Mean %TBWL
6 months	**48.04**	**15.66**
12 months	**53.09**	**17.56**
18 months	**57.98**	**16.25**
24 months	**46.57**	**15.2**
36 months	**53.18**	**14.07**
60 months	**45.3**	**15.9**

These results demonstrate significant weight loss following ESG.

*Safety:* The pooled serious adverse event (SAE) rate was 1.25%. This low rate of SAEs indicates that ESG is a relatively safe procedure.

*Quality of Evidence:* The quality of evidence from the included observational studies was assessed as very low, primarily due to the inherent limitations associated with observational study designs, such as potential biases and lack of randomization. In contrast, the quality of evidence from the single randomized controlled trial was rated as MODERATE, reflecting a more robust study design that provides a higher level of evidence despite some limitations.

**Conclusions:**

The IFSO Bariatric Endoscopy Committee, after conducting a comprehensive systematic review and meta-analysis, endorses endoscopic sleeve gastroplasty (ESG) as an effective and valuable treatment for obesity. ESG is particularly beneficial for patients with class I and II obesity, as well as for those with class III obesity who are not suitable candidates for metabolic bariatric surgery. ESG provides significant weight loss outcomes and demonstrates a favorable safety profile with a low rate of serious adverse events. Despite the limitations of the included observational studies, the randomized controlled trial included in the analysis reinforces the efficacy and safety of ESG and provides an evidence-based foundation for the position statement. Thus, the IFSO position statement supports and provides an evidence base for the role of ESG within the broader spectrum of obesity management.

## Introduction

Obesity rates are galloping, though regional, cultural, and socioeconomic factors contribute to disparities in distribution, prevalence, and incidence across the globe [1]. Still, the World Health Organization (WHO) estimates that 1.9 billion people are overweight, with 650 million having obesity as of 2016 [2]. In the United States, around 40% of the population currently live with obesity [3, 4], and prediction models estimate that this number will increase to 51% by 2030 [5]. After unsuccessful non-invasive therapies, metabolic and bariatric surgery (MBS) is the gold-standard treatment to address moderate to severe obesity. Most recently, it has also been proposed for mild obesity if it is associated with refractory metabolic diseases [6]. MBS is effective and safe in the short and long term, promoting sustained weight loss and reliable reduction in all-cause mortality rates [7].

Data show that MBS procedures have increased over decades [8, 9]. However, the rate of obesity growth is outpacing the growth in surgical interventions [10]. In addition, several patients refuse surgical treatment, others are at high surgical risk, and some suffer from overweight or mild obesity but are still not eligible for MBS. Nevertheless, obesity-related complications increase in states of overweight and mild obesity [11]. Altogether, a gap between the needs of patients with obesity and what we can offer in terms of medical and surgical interventions exists.

**Fig. 1:**
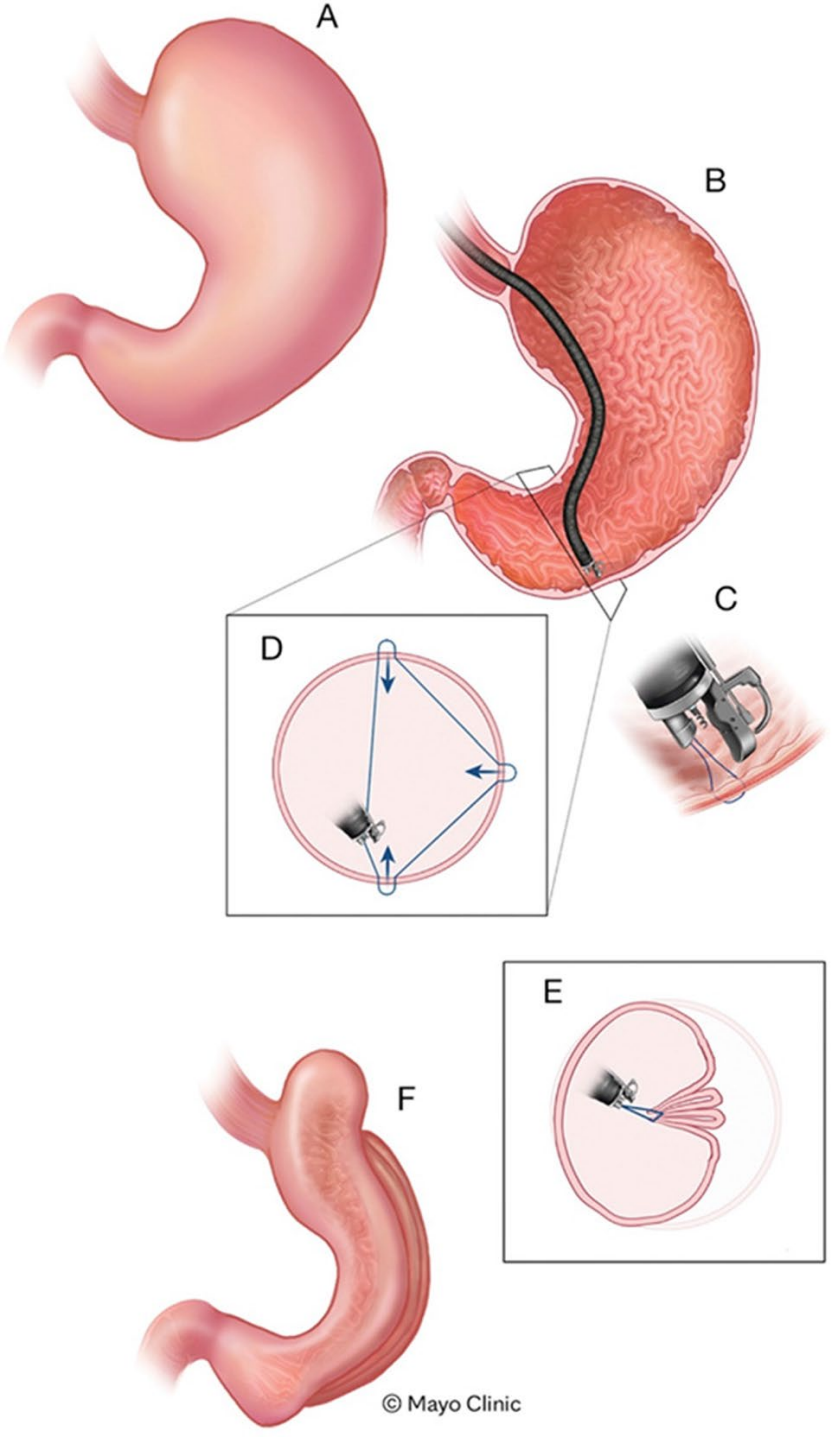
Schematic representation of the endoscopic sleeve gastroplasty procedure

This unmet need has driven the development of endoscopic solutions to address obesity, particularly when MBS is not feasible or indicated. Endoscopic bariatric therapies offer several advantages, including organ preservation, an improved risk profile, reduced healthcare utilization, and decreased burden of compliance on the patient. These benefits potentially enable the scalability of procedural offerings to effectively combat excess adiposity. Endoscopic sleeve gastroplasty (ESG) is one such solution that has gained global adoption from patients and providers in the past few years. In its current clinically adopted and regulatory approved form (Fig. 1) [12], ESG employs the Apollo Overstitch™ platform (Boston Scientific, Marlborough, MA, USA)—a full-thickness endoscopic suturing device to create apposition of the anterior against the posterior wall of the stomach, passing through the greater curvature [13, 14]. The Overstitch™ platform is currently the only US FDA–approved endoscopic suturing device for an obesity indication. Suturing starts at the transition between the gastric body and antrum, moving proximally toward the fundus, which is typically partially reduced with the preservation of a small pouch to allow fundal accommodation. Thus, it tubularizes the gastric body, altering satiety and satiation [15]. Although different stitching patterns have been proposed and discussed [16–18], the above-mentioned anatomic principles are consistent across centers and providers; thus, the procedure is clinically mature, homogeneous, and reproducible [19].

The evidence concerning the efficacy and safety of the ESG has been mounting. More than 200 international medical articles have been published on this topic, with study designs varying from large cases series [20] to cohort studies [21] and, more recently, also includes an open-label, multicenter randomized trial with 24 months follow-up [22]. The procedure is currently employed clinically in all continents, and more than 40,000 clinical procedures have been performed to date. A recent IFSO International Delphi consensus conference highlighted that multidisciplinary experts agree on the utility of ESG for managing obesity in patients with class I and II obesity and for those with class III obesity who do not wish to pursue or qualify for MBS in the context of a comprehensive multidisciplinary obesity program [23]. This IFSO Bariatric Endoscopy Committee position statement aims to augment these consensus statements by providing a comprehensive systematic review of the evidence and delivering an evidence-based position on the value of ESG within the spectrum of obesity care.

## Methods

This position statement is derived after a comprehensive systematic review to retrieve all available data on the outcomes of ESG. All the Preferred Reporting Items for Systematic Reviews and Meta-analyses (PRISMA) [24] and Cochrane Handbook for Systematic Reviews of Interventions [25] guidelines were rigorously followed for this position statement’s systematic review and meta-analysis portion. Two independent researchers (VOB and NJ) conducted all literature searches and a third independent reviewer adjudicated discrepancies. After defining the eligibility criteria, final inclusion was determined by consensus with two additional researchers (RK and BAD). One researcher collected data from the included studies using a standardized shared spreadsheet, and another independently validated the data extraction. Methodologists’ names and affiliations are in the “Acknowledgements” section.

The risk of bias in the included studies was assessed using the Joanna Briggs Institute Critical Appraisal checklist for case series [26], the New-Castle Ottawa scale for cohort studies [27], and both JADAD score [28] and a modified Cochrane Collaboration Risk of Bias tool (available from https://www.ncbi.nlm.nih.gov/books/NBK132494/bin/appf-fm1.pdf).

We used the Review Manager (Version 5.4, the Cochrane Collaboration, 2020) for pooling comparative data and the Comprehensive Meta-analysis software (Version 4, Biostat, Englewood, NJ, USA, 2022) to pool non-comparative data. Means and standard deviations (SDs) were estimated from medians and ranges based on previously validated mathematical formulas [29]. The estimation of standard deviation based on interquartile ranges, or 95% confidence interval (CI), followed the instructions in the Cochrane Handbook (Chapter 06, Section 6–5-2) [25]. If the article did not provide any measure for dispersion or sample size, we attempted to obtain them by emailing the authors. If unsuccessful, we proceeded with data input based on the SD of articles with similar sample sizes and time points (per Cochrane Handbook’s guidance).

Continuous variables were expressed preferably as means and standard deviation, while categorical ones were expressed as rates or frequencies. A *p*-value < 0.05 was considered statistically significant for a 95%CI. As a measure of effect, we employed main difference (MD) with fixed-effect mode analysis to compare data. Then, we assessed for heterogeneity among studies with the Higgins test (*I*^2^). *I*^2^ higher than 50% indicated high heterogeneity, and sensitivity analyses utilizing forest plots were undertaken to assess for outliers. If no true outliers were identified, the heterogeneity was considered true, and we switched from fixed to random-effect mode analysis to mitigate its impact on the summary estimate.

Using the results from the critical appraisal/risk of bias assessment and the meta-analysis, we evaluated the quality of the current evidence using the Grading of Recommendations, Assessment, Development, and Evaluations (GRADE) approach [30]. This standardized methodology analyzes data per outcome and uses several aspects of the studies (study design, risk of bias, imprecision, inconsistency, indirectness, publication bias, magnitude of effect, dose–response gradient, impact of residual confounding on the summary estimate) to classify the quality of the pooled evidence into 4 different categories: VERY LOW, LOW, MODERATE, and HIGH. This assessment demonstrates our certainty on how close the actual effect is to the effect estimated in our meta-analysis. All the data was input into the GRADEpro GDT online software (GRADEpro Guideline Development Tool, McMaster University, and Evidence Prime, 2022) for analysis and generation of the overall quality of evidence.

Finally, considering all the information gathered from the systematic literature review and meta-analysis, balancing the benefits and harms of the therapy, clinicians’ values and preferences, resource utilization, and cost-effectiveness, the committee determined the final position statement and level of support.

## Results

### Safety and Efficacy of ESG

#### Outcomes of ESG

##### Systematic Review

Two independent researchers (VOB and NJ) ran separate literature searches assessing eligible studies. We searched MEDLINE (PubMed), EMBASE, and gray literature from January 1, 2013 (the year ESG was described), to October 1, 2022. The final strategy was as follows:

- MEDLINE (PubMed): (total weight loss) OR (total body weight loss) OR (excess weight loss) OR (absolute weight loss) OR (excess body weight loss) OR (responders rate) OR (adverse event) OR (BMI reduction) OR (BMI decrease) OR (complication) AND (endoscopy) OR (endoscopic) OR (transoral*)OR (peroral*)OR (incisionless) AND (sleeve) OR ( overstitch) OR (gastroplasty) OR (gastric plication) OR (gastric imbrication) AND (overweight) OR (obesity) AND ("2013/01/01"[Date—Publication]: "3000"[Date—Publication])
- *EMBASE: endoscopic AND sleeve AND gastroplasty OR (apollo AND overstitch) AND [embase]/lim NOT ([embase]/lim AND [medline]/lim) AND ('article'/it OR 'article in press'/it OR 'conference review'/it OR 'note'/it OR 'review'/it)*

The eligibility criteria included:

- Articles published online from 1 Jan. 2013 until 1 Oct. 2022 (last search update);
- ESG performed with the Apollo Overstitch device (no restriction as to stitching pattern);
- No language restriction;
- Full-text articles only;
- Study designs case series with sample ≥ 10, cohort studies, case–control studies, and randomized trials. For the non-comparative meta-analysis, we extracted results from the ESG cohort from comparative studies;
- To avoid overestimating the real sample, only the most recent or the most representative (larger sample) study was considered for each center if repeated data was suspected;
- Studies describing outcomes at predetermined time points: 6, 12, 18, 24, 36, > 36 months;
- Studies reporting efficacy and/or safety data.

**Fig. 2:**
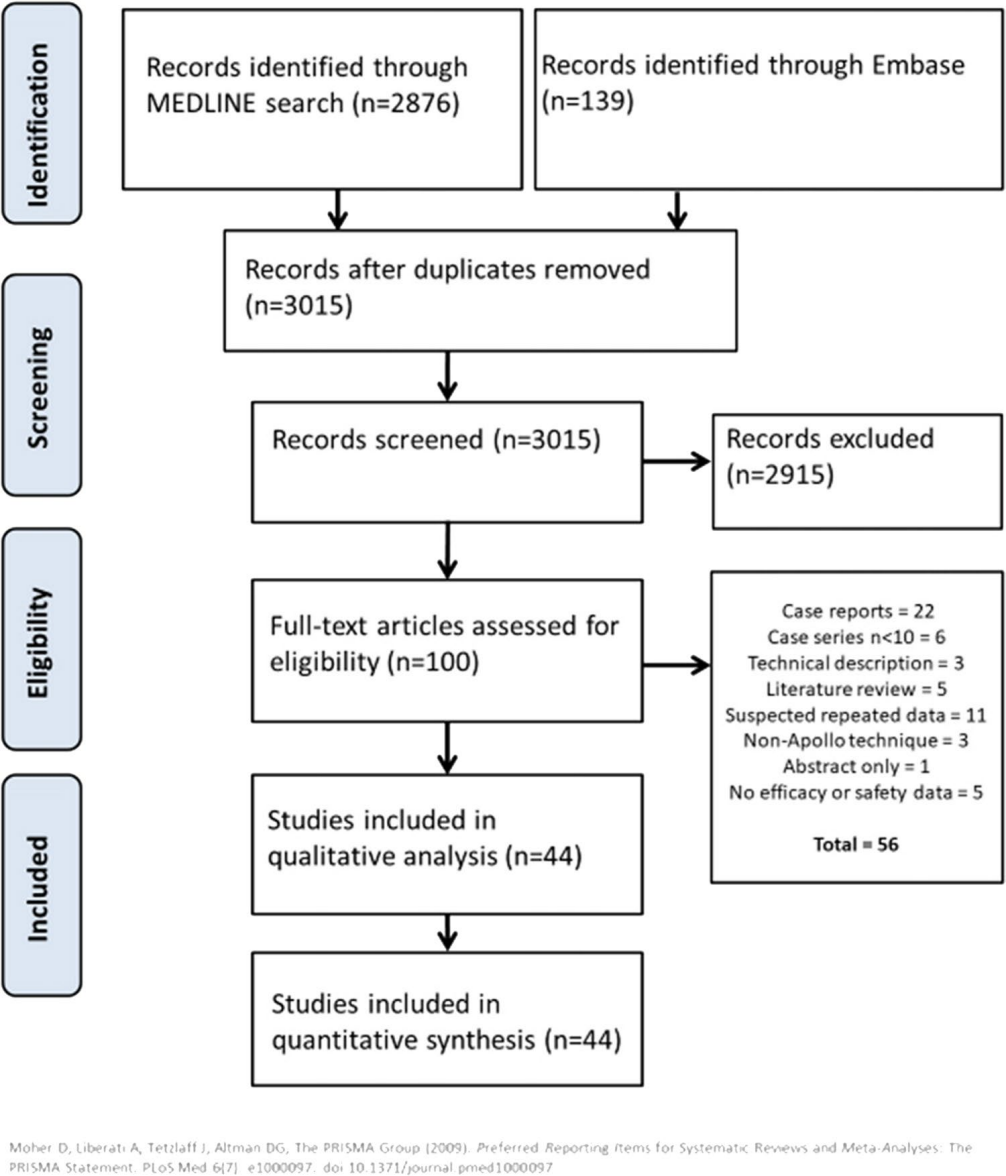
PRISMA flowchart for the literature screening and inclusion/exclusion process for the overall outcomes of ESG (non-comparative analysis)

The initial search retrieved 3015 records. After screening titles and abstracts, 100 articles were selected for full-text assessment. Finally, 44 articles were included in the qualitative and quantitative analyses. Figure 2 shows the screening and inclusion/exclusion flowchart.

##### Descriptive Analysis

- **Baseline and Demographic Data**
  Among the 44 articles, we identified 29 case series, 14 cohort studies, and 1 randomized controlled trial (RCT). Among the cohort studies, 7 compared ESG to LSG, 1 compared ESG to lifestyle intervention alone, 1 compared ESG versus ESG plus anti-obesity medication (liraglutide), 2 compared ESG and intragastric balloons, and 3 compared ESG cohorts with different stitching patterns. Table 1 summarizes the overall and baseline data of the qualitative analysis of the included studies.

**Table 1:** Baseline data of the 44 articles included in the meta-analysis of the ESG outcomes

Author (year)	Study design	Single vs. multicenter	Retrospective vs. prospective	Population (total sample)	Intervention (*n*)	Comparison (*n*)	Observations	Inclusion criteria (BMI)	Age	Sex	Mean baseline BMI (kg/m2)	Type II Diabetes
Abu Dayyeh, B. K., et al. [31]	Case series	Single center	Prospective	25	ESG	-	Study of gastric physiology	BMI > 30 and < 40	47.6 (10)	21 women	35.5 (2.6)	1
Abu Dayyeh, B. K., et al. [22]	Randomized clinical trial	9 centers	Prospective	209	ESG (85)	Lifestyle intervention (124)	Open-label FDA-regulated trial	BMI > 30 and < 40	47.3 (9.3) ESG and 45.7 (10) lifestyle intervention	68 (88%) ESG and 91 (84%) lifestyle intervention	35.5 (2.6) ESG and 35.7 (2.6) lifestyle intervention	18 ESG and 36 lifestyle intervention
Alqahtani, A., et al. [20]	Case series	Single center	Prospective	1000	ESG	-	-	Ineligible for or refuse bariatric surgery	34.4 (9.5)	897 (89.7) women	33.3 (4.5)	17
Alqahtani, A., et al. [32]	Case series	Single center	Prospective	109	ESG	-	Patients aged < 21 years old	BMI > 120% of the 95th percentile	17.6 (2.2)	99 (91.7) women	33 (4.7)	NR
Alqahtani, A. R., et al. [33]	Cohort (propensity score-matched)	Single center	Retrospective	6036	ESG (3018)	LSG (3018)	-	BMI > 27.5	33.8 (9.6) ESG and 33.9 (9.7) LSG	2686 (89%) women for both groups	32.5 (3.1) ESG and 32.9 (3.5) LSG	112 ESG
Asokkumar, R., et al. [34]	Case series	Single center	Prospective	35	ESG	-	-	BMI > 27.5	43.6 (11.3)	20 (52.7%) women	34 (4.9)	8 (23%)
Badurdeen, D., et al. [35]	Cohort (propensity score-matched)	3 centers	Prospective	52	ESG (26)	ESG + Liraglutide (26)	-	BMI > 27	41.15 (10.64) ESG and 40.65 (8.69) ESG + Liraglutide	16 women ESG and 17 women ESG + Liraglutide	35.56 (1.68) ESG and 35.83 (2.33) ESG + Liraglutide	16 (8 and 8)
Barrichello, S., et al. [36]	Case series	7 centers	Retrospective	193	ESG	-	-	BMI > 25	42.3 (9.6)	148 women	34.11 (2.97)	NR
Bhandari, M., et al. [37]	Case series	Single center	Retrospective	53	ESG	-	-	BMI > = 28	40.54 (13.79)	43 (81.1%)	34.78 (5.20)	10
Callahan, Z. M., et al. [38]	Case series	Single center	Retrospective	10	ESG	-	Study on GI tract suturing including 10 ESG cases	NR	50.2 (12.2)	10 women	NR	NR
Carr, P., et al. [39]	Cohort	Single center	Prospective	61	ESG (16)	LSG (45)	-	BMI > 26 with comorbidity or BMI > 30	41.4 (10.4) vs 40.4 (9.0)	31 women (57.4%) ESG and 59 (71.1%) LSG	35.5 (5.2) vs 40.7 (5.6)	0 ESG and 2 LSG
Cheskin, L. J., et al. [40]	Cohort (propensity score-matched)	Single center	Retrospective	386	ESG (105)	Lifestyle intervention (281)	-	NR	47.58 (11.97) ESG and 48.17 (12.18) Lifestyle intervention	75 (71.42%) women ESG and 189 (67.2%) women Lifestyle intervention	40.5 (7.89) ESG and 39.85 (7.62) lifestyle intervention	NR
Espinet-Coll, E., et al. [41]	Case series	Single center	Prospective	38	ESG	-	Focus on persistence of sutures	BMI > 27	47 (5.5)*	30 women	37.6 (3)*	NR
Espinet-Coll, E., et al. [42]	Cohort	2 centers	Retrospective	88	ESG (standard stitching pattern = "TBp")	ESG with different stitching patterns ("Lp" and "TMp")	Comparison of 3 different stitching patterns	BMI > 27	Overall 46.1 (12.3)	61 women	Overall 39.40 (4.69)	Overall 11 (12.5%)
Farha, J., et al. [43]	Cohort	2 centers	Retrospective	247	ESG (98)	ESG with fundal suturing (149)	-	BMI > 30	44.9 (9.4) vs 47.2 (11.5)	84 (85.7%) and 107 (71.8%)	38.3 (5.6) vs 39.4 (7.3)	Overall 16
Fayad, L., et al. [44]	Cohort	Single center	Retrospective	137	ESG (54)	LSG (83)	-	NR	48 (12) ESG and 47.75 (6.16) LSG*	31 women (57.4%) ESG and 59 (71.1%) LSG	43.07 (8.85) ESG and 44.12 (5.78) LSG*	3.7% ESG and 20.48% LSG
Fayad, L., et al. [45]	Cohort	Single center	Retrospective	105	ESG (58)	IGB (47)	-	BMI > 27 for IGB and BMI > 30 for ESG	48.2 (11.8) ESG and 47.7 (12.4) IGB	34 women (58.6%) ESG and 46 (97.9%) IGB	41.5 (8.2) ESG and 34.5 (6.7) IGB	3 (5.2) ESG and 4 (8.5) IGB
Fiorillo, C., et al. [46]	Cohort	Single center	Retrospective	46	ESG (23)	LSG (23)	Focus on quality of life after 6 months	NR	41 (2) ESG and 37 (4.5) LSG*	16 women ESG and 17 LSG	39.5 (2) ESG and 41 (1.27) LSG*	2 ESG and 3 LSG
Ghoz, H., et al. [47]	Case series	Single center	Retrospective	20	ESG	-	Focus on nutritional deficiencies	NR	46.2 (14.1)	17 women	36.4 (4.1)	2
Glaysher, M. A., et al. [48]	Cohort	Single center	Prospective	32	ESG without longitudinal compression (9)	ESG with longitudinal compression (23)	-	BMI > 30	45 (12) ESG and 43 (10) ESG with compression	5 women ESG and 18 ESG with compression	36.62 (3.72) ESG and 36.42 (3.27) ESG with compression*	NR
Graus Morales, J., et al. [49]	Case series	Single center	Prospective	148	ESG	-	ESG with modified stitching pattern	BMI > 30	41.53 (10)	121 women	35.11 (5.5)	NR
Gudur, A. R., et al. [50]	Cohort	Multicenter (database)	Retrospective	36,323	ESG (6,053)	LSG (30,270)	MBSAQIP database cohort study	-	47.47 (11.44) and 44.87 (11.94)	5116 and 24926 women	40.54 (8.65) and 42.8 (6.17)	1040 and 5775
Hajifathalian, K., et al. [51]	Case series	Single center	Prospective	118	ESG	-	Focus on NAFLD scores	BMI > 30 and NAFLD	46 (13)	80 (68%) women	40 (7)	35
Hill, C., et al. [52]	Case series	Single center	Prospective	21	ESG	-	Focus on learning curve	NR	47.7 (11.2)	13 women	41.8 (8.5)	NR
Jagtap, N., et al. [53]	Case series	Single center	Prospective	26	ESG	-	Focus on NAFLD scores	BMI > 27.5 and NAFLD	41.5 (9.58)	16 women	36.55 (5.07)	13
James, T. W., et al. [54]	Case series	Single center	Retrospective	100	ESG	-	Non-academic setting	NR	45 (9)	86 women	38.41 (5.44)	4
Kumar, N., et al. [55]	Case series	Multicenter (NS)	Prospective	122	ESG (23 in phase 1, 22 in phase 2, 77 in phase 3)	-	Focus on technical refinement over time	BMI > 30	Phase 1 37.7 (1.9); Phase 2 39.2 (1.6); Phase 3 41.3 (1.1)	Phase 1 19 women; phase 2 20 women; phase 3 59 women	Phase 1 34.2 (1.1); Phase 2 34.3 (1); Phase 3 36.1 (0.6)	NR
Li, R., et al. [56]	Case series	Single center	Prospective	24	ESG	-	Focus on high-risk cases	BMI > 50, severe comorbidities, or impenetrable abdomen	55.6 (9.2)	6 women	49.9 (14.4)	15
Lopez-Nava, G., et al. [57]	Case series	Single center	Retrospective	435	ESG	-		BMI > 30	48.5 (10.2)	314 women	38.9 (5.3)	NR
Lopez-Nava, G., et al. [58]	Cohort	2 centers	Prospective	24	ESG (12)	LSG (12)	Focus on enterohormonal changes	BMI > 30	49.3 (2.4) ESG and 50.5 (1.9) LSG	9 women ESG and 9 women LSG	38.3 (1.8) ESG and 39.2 (1.5) LSG	0 ESG and 3 preDM LSG
Lopez-Nava, G., et al. [59]	Case series	3 centers	Prospective	248	ESG	-	-	NR	44.5 (10)	181 women	37.8 (5.6)	NR
Manos, T., et al. [60]	Case series	Single center	Retrospective	191	ESG	-	Single-channel endoscope device (Overstitch SX)	BMI > 30	36.9 (no SD)	173 women	33.7 (4.18)*	NR
Matteo, M. V., et al. [61]	Case series	Single center	Prospective	18	ESG	-	Patients > 65 years old	BMI > 30	67 (4.5)	10 women	41.2 (5.9)	4
Maydeo, A., et al. [16]	Case series	Single center	Prospective	58	ESG	-	Different stitching pattern ("accordion")	BMI > 28	42.1 (8.7)	55 women	37.88 (5.76)	17
Mehta, A., et al. [62]	Case series	Single center	Prospective	50	ESG	-	Focus on quality of life and mental health	NR	49.5 (14)	37 women	38.5 (5.8)	9
Neto, M. G., et al. [63]	Case series	4 centers	Prospective	233	ESG	-	-	BMI > 30 and < 40	41.1 (10.5)	170 women	34.7 (2.6)	12
Neto, M. G., et al. [64]	Case series	Multicenter (NS)	Retrospective	1828	ESG	-	Clinical consensus gathering 47 Brazilian endoscopists	NR	NR	NR	NR	NR
Novikov, A. A., et al. [65]	Cohort	Single center	Retrospective	278	ESG (91)	LSG (120) and LAGB (67)	-	BMI > 30	43.86 (11.26) ESG, 40.71 (11.95) LSG, and 41.94 (13.31) LAGB	62 women ESG, 94 women LSG, and 54 women LAGB	38.61 (6.98) ESG, 47.22 (7.84) LG, and 44.98 (6.45) LAGB	20 ESG, 31 LSG, and 15 LAGB
Pizzicannella, M., et al. [66]	Case series	Single center	Prospective	133	ESG	-	Focus on durability of sutures and their correlation with weight loss	NR	NR	NR	43.2 (8.6)	NR
Rapaka, B., et al. [67]	Cohort	2 centers	Prospective	41	ESG (23)	IGB (18)	-	NR	47.69 (5.06) ESG and 41.06 (8.81) IGB	20 women ESG and 18 IGB	41.21 (5.38) ESG and 34.5 (4.46)	NR
Sarkar, A., et al. [68]	Case series	6 centers	Retrospective	91	ESG	-	Focus on new bariatric endoscopy programs	BMI > 30	39.7 (11.6)	56 women	38.7 (4.4)*	46
Sartoretto, A., et al. [69]	Case series	3 centers	Retrospective	112	ESG	-	-	BMI > 27	45.1 (11.7)	77 women	37.9 (6.7)	14
Saumoy, M., et al. [70]	Case series	Single center	Prospective	128	ESG	-	Focus on learning curve	BMI > 30	43.62 (11.37)	86 women	38.92 (6.95)	NR
Sharaiha, R. Z., et al. [71]	Case series	Single center	Retrospective	216	ESG	-	Long-term follow-up	BMI > 30 or > 27 with comorbidities	46 (13)	146 women	39 (6)	67

*Calculated field
  Among the 44 articles, the total sample included 49,848 patients (15,714 ESG and 34,134 controls, including LSG, laparoscopic adjustable gastric banding, and IGB). At baseline, the mean age and BMI were 44.24 (SE 1.405, 95%CI 41.48–46–99, 41 articles *n* = 13,562) and 37.56 (SE 0.45 95%CI 36.66–38.46, 42 articles, *n* = 13,876), respectively. Most patients were female (11,449 females, 83.2% and 2304 males, 16.8%, 42 articles, *n* = 13,753).

##### Risk of Bias/Critical Appraisal Assessment

All included studies were assessed for their risk of bias using specific tools based on the study design. Case series were evaluated using the Joanna Briggs Institute Critical Appraisal Checklist. Ten items are scored based on the perceived risk, and the scoring is positive. The scale ranges from 0 to 10, with 0 being the highest risk of bias and 10 being the lowest. The included case series (29 articles) had a mean score of 7.5 ± 1.8. Reporting of outcomes, and follow-up, and statistical analyses were the two topics with the worst positive scoring (16/29, 55.2%).

For cohort studies, we employed the New-Castle Ottawa scale that assesses 8 topics for bias. The scale ranges from 0 to 9, with 0 being the highest risk of bias and 9 being the lowest. The 14 included cohort articles scored an average of 6.07 ± 1.43. The selection of a non-exposed cohort and the duration of follow-up were the two topics with the worst scoring, thus most subject to bias (0.42 ± 0.51 and 0.35 ± 0.49).

For the single RCT [22], the JADAD score was 3, which is the maximum score for open-label trials. As to the modified Cochrane risk of bias tool, the trial was at low risk for selection and reporting bias. However, we detected a high risk of other biases: performance, detection, and attrition. The GRADE assessment of the quality of evidence later weighted the impact of those biases. Tables 2, 3, and 4 summarize the assessment of biases for case series, cohorts, and RCT, respectively.

**Table 2:** Critical appraisal and risk of bias assessment for the included case series

CASE SERIES													
Author, year	Study design	JBI Critical appraisal checklist domains											
Articles (n) = 29		*Were there clear criteria for inclusion in the case series*	*Was the condition measured in a standard, reliable way for all participants included in the case series?*	*Were valid methods used for identification of the condition for all participants included in the case series?*	*Did the case series include consecutive participants?*	*Did the case series have complete inclusion of participants?*	*Was there clear reporting of the demographics of the participants in the study?*	*Was there transparent reporting of clinical information of the participants?*	*Were the outcomes or follow−up results of cases clearly reported?*	*Was there transparent reporting of the demographic information of the presenting site(s)/clinic(s)?*	*Was statistical analysis appropriate?*	*Obs.*	*Total of "yes" (max=10)*
Abu Dayyeh, B. K., et al. [31]	Case series	yes	yes	yes	yes	yes	yes	yes	no	yes	no		*8*
Alqahtani, A., et al. [20]	Case series	yes	yes	yes	yes	yes	yes	yes	no	yes	no		*8*
Alqahtani, A., et al. [32]	Case series	yes	yes	yes	yes	yes	yes	yes	yes	yes	no	Pediatric population	*9*
Asokkumar, R., et al. [34]	Case series	yes	yes	yes	yes	yes	yes	yes	no	yes	no		*8*
Barrichello, S., et al. [36]	Case series	yes	yes	yes	yes	yes	yes	yes	no	yes	no		*8*
Bhandari, M., et al. [37]	Case series	yes	yes	yes	yes	yes	yes	yes	no	yes	no		*8*
Callahan, Z. M., et al. [38]	Case series	yes	yes	yes	yes	yes	yes	yes	no	yes	no		*8*
Espinet−Coll, E., et al. [41]	Case series	yes	yes	yes	yes	yes	yes	no	no	yes	no		*7*
Ghoz, H., et al. [47]	Case series	yes	yes	yes	yes	yes	yes	no	no	no	no		*6*
Graus Morales, J., et al. [17, 49]	Case series	yes	yes	yes	yes	yes	yes	yes	no	yes	no		*8*
Hajifathalian, K., et al. [51]	Case series	yes	yes	yes	yes	yes	yes	yes	no	yes	no		*8*
Hill, C., et al. [52]	Case series	yes	yes	yes	yes	yes	yes	yes	no	yes	no	Only for adverse events, no efficacy data	*8*
Jagtap, N., et al. [53]	Case series	yes	yes	yes	yes	yes	yes	yes	no	yes	yes		*9*
James, T. W., et al. [54]	Case series	yes	yes	yes	yes	yes	yes	yes	yes	yes	yes		*10*
Kumar, N., et al. [55]	Case series	yes	unclear	unclear	no	unclear	no	no	yes	no	yes		*3*
Li, R., et al. [56]	Case series	yes	yes	yes	no	unclear	yes	yes	yes	yes	yes		*8*
Lopez−Nava, G., et al. [57]	Case series	yes	yes	yes	unclear	no	yes	no	yes	yes	yes		*7*
Lopez−Nava, G., et al. [59]	Case series	yes	yes	yes	yes	unclear	yes	unclear	yes	yes	yes		*8*
Manos, T., et al. [60]	Case series	yes	yes	yes	unclear	unclear	yes	no	yes	no	yes		*6*
Matteo, M. V., et al. [61]	Case series	yes	yes	yes	yes	yes	yes	yes	yes	yes	yes		*10*
Maydeo, A., et al. [16]	Case series	yes	yes	yes	unclear	unclear	yes	yes	unclear	yes	yes		*7*
Mehta, A., et al. [62]	Case series	unclear	unclear	yes	unclear	unclear	yes	yes	yes	yes	yes		*6*
Neto, M. G., et al. [63]	Case series	yes	yes	yes	unclear	unclear	yes	yes	yes	yes	yes		*8*
Neto, M. G., et al. [64]	Case series	no	unclear	unclear	unclear	unclear	no	no	yes	no	unclear	Only for adverse events, no efficacy data	*1*
Pizzicannella, M., et al. [66]	Case series	yes	yes	yes	yes	yes	no	no	yes	yes	yes		*8*
Sarkar, A., et al. [68]	Case series	yes	yes	yes	unclear	unclear	yes	yes	yes	no	yes		*7*
Sartoretto, A., et al. [69]	Case series	yes	yes	yes	yes	unclear	yes	yes	yes	yes	yes		*9*
Saumoy, M., et al. [70]	Case series	yes	yes	yes	yes	yes	yes	no	yes	yes	yes		*9*
Sharaiha, R. Z., et al. [71]	Case series	yes	yes	yes	yes	yes	yes	yes	yes	yes	yes		*10*
Summary		*Yes = 27 (93.1%)* *No = 1 (3.4%)* *Unclear = 1 (3.4%)*	*Yes = 26 (89.7%)* *No = 0* *Unclear = 3 (10.3%)*	*Yes = 27 (93.1%)* *No = 0* *Unclear = 2 (6.9%)*	*Yes = 27 (93.1%)* *No = 1 (3.4%)* *Unclear = 1 (3.4%)*	*Yes = 18 (62.1%)* *No = 1 (3.4%)* *Unclear = 10 (34.5%) *	*Yes = 26 (89.6%)* *No = 3 (10.4%)* *Unclear = 0*	*Yes = 20 (69%)* *No = 8 (27.6%)* *Unclear = 1 (3.4%)*	*Yes = 16 (55.2%)* *No = 12 (41.4%)* *Unclear = 1 (3.4%)*	*Yes = 24 (82.8%)* *No = 5 (17.2%)* *Unclear = 0*	*Yes = 16 (55.2%)* *No = 12 (41.4%)* *Unclear = 1 (3.4%)*		*7.5 +/− 1.89*

**Table 3:** Critical appraisal and risk of bias assessment for the included cohort studies

COHORT STUDIES										
Author, year	Study design	New Castle-Ottawa Scale for cohort studies domains								
		*Representativeness of the exposed cohort (1)*	*Selection of the non-exposed cohort (1)*	*Ascertainment of exposure (1)*	*Demonstration that outcome of interest was not present at start of study (1) *	*Comparability of cohorts based on the design or analysis (2)*	*Assessment of outcome (1)*	*Was follow-up long enough for outcomes to occur (1)*	*Adequacy of follow-up of cohorts (1) *	*Total score (max = 9)*
Alqahtani, A. R., et al. [33]	Cohort	1	0	0	1	1	1	0	1	5
Badurdeen, D. et al. [35]	Cohort	1	0	0	1	0	1	0	1	4
Carr, P., et al. [39]	Cohort	1	0	0	1	1	1	0	1	5
Cheskin, L. J., et al. [40]	Cohort	1	0	1	1	1	1	0	1	6
Espinet-Coll, E., et al. [41]	Cohort	1	0	1	1	1	1	0	1	6
Farha, J., et al. [43]	Cohort	1	1	0	1	1	1	1	1	7
Fayad, L., et al. [44]	Cohort	1	1	0	1	1	1	0	1	6
Fayad, L., et al. [45]	Cohort	1	0	0	1	1	1	0	1	5
Fiorillo, C., et al. [46]	Cohort	1	0	1	1	1	1	0	1	6
Glaysher, M. A., et al. [48]	Cohort	1	1	1	1	1	1	1	1	9
Gudur, A. R., et al. [50]	Cohort	1	1	1	1	1	1	1	1	*9*
Lopez-Nava, G., et al. [58]	Cohort	1	1	1	1	1	1	1	1	9
Novikov, A. A., et al. [65]	Cohort	1	1	1	1	0	1	1	0	6
Rapaka, B., et al. [67]	Cohort	1	1	1	1	0	1	1	0	6
***Summary***		**1 (0)**	**0.42 (0.51)**	**0.5 (0.51)**	**1 (0)**	**0.78 (0.42)**	**1 (0)**	**0.35 (0.49)**	**0.85 (0.36)**	***6.07 (1.43)***

**Table 4:** Critical appraisal and risk of bias assessment for the included randomized clinical trial

RANDOMIZED TRIALS												
Author, year	Study design	Cochrane risk of bias tool							JADAD score			
		*Selection bias: Random Sequence generation*	*Selection bias: Allocation Concealment*	*Reporting Bias: Selective Reporting*	*Other Bias - other sources of bias*	*Performance bias*	*Detection bias*	*Attrition bias*	*Randomization*	*Blinding*	*Withdrawals*	*Total*
Abu Dayyeh, B. K., et al. [22]	Randomized clinical trial	Low	Low	Low	Low	High	High	High	2	0	1	3

##### Meta-analysis

Forty-two articles reported %Excess Weight Loss (%EWL) and/or %Total Body Weight Loss (%TBWL) at least in one time point of interest (6, 12, 18, 24, 36, > 36 months). Two articles [50, 64] only reported safety outcomes. Continuous variables (%EWL and %TBWL) were pooled using the CMA software, and the results are presented ahead of time according to time points. Categorical variables were pooled using absolute numbers to calculate pooled rates. Four articles reported the responder rate as ≥ 5%TWL at 12 months, and 9 reported it as ≥ 10%TBWL. The pooled rates were 422/478 (88.3%) and 632/768 (82.3%). Forty articles reported the SAEs rate (according to the FDA definition from https://www.fda.gov/safety/reporting-serious-problems-fda/what-serious-adverse-event). Among 15,398 ESG procedures, 194 events fulfilled the criteria for SAE for a pooled rate of 1.25%. Table 5 shows all outcomes of the included studies according to follow-up time points, and Fig. 2 graphically depicts weight loss outcomes over time.

**Table 5:** Outcomes of the studies included in the non-comparative meta-analysis

Author (year)	Population (total sample)	Intervention (n)	Comparison (n)	%EWL	n	6 months	n	12 months	n	18 months	n	24 months	n	≥36 months	%TBWL
Abu Dayyeh, B. K., et al. [31]	25	ESG	−		25	53 (17)	10	54 (40)	8	45 (41)	−	−	−	−	
Abu Dayyeh, B. K., et al. [22]	209	ESG (85)	Lifestyle intervention (124)		−	−	77	49.2 (32)	−	−	50	41 (32)	−	−	
Alqahtani, A., et al. [20]	1000	ESG	−		369	64.3 (56.2)	216	67.5 (52.3)	54	64.7 (55.4)	−	−	−	−	
Alqahtani, A., et al. [32]	109	ESG	−		82	80.1 (63.3)	43	87.1 (59.5)	24	70.9 (55.5)	17	63.8 (52.3)			
Alqahtani, A. R., et al. [33]	6036	ESG (3018)	LSG (3018)		2490	67.0 (28.6)	2243	77.1 (24.6)	−	−	1911	75.2 (47.9)	854	59.7 (57.1)	
Asokkumar, R., et al. [34]	35	ESG	−		−	−	−	−	−	−	−	−	−	−	
Badurdeen, D. et al. [35]	52	ESG (26)	ESG + Liraglutide (26)		26	69.94 (6.3)	−	−	−	−	−	−	−	−	
Barrichello, S., et al. [36]	193	ESG	−		181	56.15 (22.93)	121	59.41 (25.69)	−	−	−	−	−	−	
Bhandari, M., et al. [37]	53	ESG	−		−	−	−	−	−	−	−	−	−	−	
Callahan, Z. M., et al. [38]	10	ESG	−		−	−	3	17.6 (47.3)	−	−	4	12.7 (16.9)	−	−	
Carr, P., et al. [39]	61	ESG (16)	LSG (45)		13	51 (11)	9	57 (32)	−	−	−	−	−	−	
Cheskin, L. J., et al. [40]	386	ESG (105)	Lifestyle intervention (281)		−	−	−	−	−	−	−	−	−	−	
Espinet−Coll, E., et al. [41]	38	ESG	−		−	−	−	48.3 (18.5)*	−	−	−	−	−	−	
Espinet−Coll, E., et al. [18]	88	ESG (standard stitching pattern = "TBp")	ESG with different stitching patterns ("Lp" and "TMp")		−	−	88	46.41 (20.6)	−	−	−	−	−	−	
Farha, J., et al. [43]	247	ESG (98)	ESG with fundal suturing (149)		82 99	54.7 (19.2) 37.7 (17.3)	57 66	65.3 (21.1) 40.6 (23.5)	−	−	−	−	−	−	
Fayad, L., et al. [44]	137	ESG (54)	LSG (83)		−	−	−	−	−	−	−	−	−	−	
Fayad, L., et al. [45]	105	ESG (58)	IGB (47)		−	−	−	−	−	−	−	−	−	−	
Fiorillo, C., et al. [46]	46	ESG (23)	LSG (23)		23	39.05 (20.7)*	−	−	−	−	−	−	−	−	
Ghoz, H., et al. [47]	20	ESG	−		−	−	−	−	−	−	−	−	−	−	
Glaysher, M. A., et al. [48]	32	ESG without longitudinal compression (9)	ESG with longitudinal compression (23)		5 7	42.4 (18.1)* 65.6 (23.9)*	−	−	−	−	−	−	−	−	
Graus Morales, J., et al. [17, 49]	148	ESG	−		148	64.93 (51)	148	75.4 (85)	72	79.25 (43)	−	−	−	−	
Gudur, A. R., et al. [50]	36,323	ESG (6,053)	LSG (30,270)		−	−	−	−	−	−	−	−	−	−	
Hajifathalian, K., et al. [51]	118	ESG	−		114	45.3 (29.39)*	100	47.8 (32.65)*	−	−	78	45.5 (33.11)*	−	−	
Hill, C., et al. [52]	21	ESG	−		−	−	−	−	−	−	−	−	−	−	
Jagtap, N., et al.[53]	26	ESG	−		26	32.67 (19.51)	26	51.33 (17.33)	−	−	−	−	−	−	
James, T. W., et al. [54]	100	ESG	−		34	48.9 (19.9)	12	66.1 (21.5)	−	−	−	−	−	−	
Kumar, N., et al. [55]	122	ESG (23 in phase 1, 22 in phase 2, 77 in phase 3)	−		−	−	−	−	−	−	−	−	−	−	
Li, R., et al. [56]	24	ESG	−		12	25 (9.1)	7	29.1 (17.9)	−	−	−	−	−	−	
Lopez−Nava, G., et al. [57]	435	ESG	−		−	−	−	−	−	−	−	−	−	−	
Lopez−Nava, G., et al. [58]	24	ESG (12)	LSG (12)		−	−	−	−	−	−	−	−	−	−	
Lopez−Nava, G., et al. [59]	248	ESG	−		−	−	−	−	−	−	−	−	−	−	
Manos, T., et al. [60]	191	ESG	−		84	41.6 (20**)	69	34.7 (22**)	−	−	−	−	−	−	
Matteo, M. V., et al. [61]	18	ESG	−		18	39.25 (5.5)*	12	38.25 (10.97)*	10	40.25 (13.26)*	10	41 (8.08)*	−	−	
Maydeo, A., et al. [16]	58	ESG	−		52	42.8 (13.1)	−	−	−	−	−	−	−	−	
Mehta, A., et al. [62]	50	ESG	−		47	46.9 (22.4)	39	50.5 (24.9)	47	47.7 (26.5)	−	−	−	−	
Neto, M. G., et al. [63]	233	ESG	−		178	47.1 (18)	123	54.8 (17.4)	−	−	−	−	−	−	
Neto, M. G., et al. [64]	1828	ESG	−		−	−	−	−	−	−	−	−	−	−	
Novikov, A. A., et al. [65]	278	ESG (91)	LSG (120) and LAGB (67)		−	−	−	−	−	−	−	−	−	−	
Pizzicannella, M., et al. [66]	133	ESG	−		87	34.5 (19.8)	41	34.3 (21.9)	−	−	−	−	−	−	
Rapaka, B., et al. [67]	41	ESG (23)	IGB (18)		23**	16.17 (5.69)	−	−	−	−	−	−	−	−	
Sarkar, A., et al. [68]	91	ESG	−		52	35.6 (20**)	−	−	−	−	−	−	−	−	
Sartoretto, A., et al. [69]	112	ESG	−		52	50.3 (22.4)	−	−	−	−	−	−	−	−	
Saumoy, M., et al. [70]	128	ESG	−		−	−	−	−	−	−	−	−	−	−	
Sharaiha, R. Z., et al. [71]	216	ESG	−		−	−	142	47.9 (33.11)*	−	−	−	−	36 months = 68 60 months = 56	45.1 (42.67)* 45.3 (47.32)*	
***Total***	**49848**	**−**	**−**		**4329**	**48.04 (SE 3.59)**	**3652**	**53.09 (SE 4.15)**	**215**	**57.98 (SE 7.38)**	**2070**	**46.57 (SE 9.85)**	**36 months = 922**	**36 months = 53.18 (SE 7.25)**	

Author (year)	n	6 months	n	12 months	n	18 months	n	24 months	n	≥36 months	12−month Responder rate (≥5%TBWL)	12−month Responder rate (≥10%TBWL)	SAEs	Obs.	
Abu Dayyeh, B. K., et al. [31]	−	−	−	−	−	−	−	−	−	−	−	−	3/25		
Abu Dayyeh, B. K., et al. [22]	−	−	77	13.6 (8.0)	−	−	50	11.4 (8.4)	−	−	70/77	48/77	6/150		
Alqahtani, A., et al. [20]	369	13.7 (6.8)	216	15 (7.7)	54	14.8 (8.5)	−	−	−	−	193/216	−	24/1000		
Alqahtani, A., et al. [32]	82	14.4 (6.5)	43	16.2 (8.3)	24	15.4 (9.2)	17	13.7(8)	−	−	−	−	1/109		
Alqahtani, A. R., et al. [33]	2490	15.1 (6.1)	2243	19.2 (7.7)	−	−	1911	16.2 (9.7)	854	14 (12.1)	−	−	14/3018		
Asokkumar, R., et al. [34]	10	16.2 (4.9)	−	−	−	−	−	−	−	−	−	−	0/35		
Badurdeen, D. et al. [35]	26	20.51 (1.68)	−	−	−	−	−	−	−	−	−	−	1/52		
Barrichello, S., et al. [36]	181	14.25 (5.26)	121	15.06 (5.22)	−	−	−	−	−	−	−	−	2/193		
Bhandari, M., et al. [37]	42	14.25 (6.17)	42	19.94 (4.89)	−	−	−	−	−	−	−	−	0	37/42 (88%) had >15% TWL at 12 months	
Callahan, Z. M., et al. [38]	−	−	−	−	−	−	−	−	−	−	−	−	2/10		
Carr, P., et al. [39]	13	15 (6)	9	18 (11)	−	−	−	−	−	−	−	−	0	ESG 78% >25% EWL and 55.6% >20%TBWL at 12months	
Cheskin, L. J., et al. [40]	63	17.7 (6.4)	43	20.6 (8.3)	−	−	−	−	−	−	41/43	39/43	5/105	28/105 presented >20% TBWL	
Espinet−Coll, E., et al. [41]	−	−	−	17.1 (3.1)*	−	−	−	−	−	−	−	36/38	1/38		
Espinet−Coll, E., et al. [18]	−	−	88	17.36 (6.09)	−	−	−	−	−	−	−	84/88	0/88		
Farha, J., et al. [43]	82 99	17.3 (4.5) 16.2 (7.0)	57 66	21.3 (6.2) 17.5 (10.2)	−	−	−	−	−	−	−	−	6/247		
Fayad, L., et al. [44]	35	17.1 (6.5)	−	−	−	−	−	−	−	−	−	−	3/54	39/54 had >15% TBWL 72.2% at 6 months	
Fayad, L., et al. [45]	25	19.5 (5.7)	21	21.3 (6.6)	−	−	−	−	−	−	−	−	3/58		
Fiorillo, C., et al. [46]	23	13.87 (6.55)*	−	−	−	−	−	−	−	−	−	−	0/23		
Ghoz, H., et al. [47]	13	13.7 (9.3)	10	16.2 (10.4)	−	−	−	−	−	−	−	−	NR		
Glaysher, M. A., et al. [48]	5 7	12.4 (3.1)* 20.5 (5)*	−	−	−	−	−	−	−	−	−	−	0/32		
Graus Morales, J., et al. [17, 49]	148	15.45 (5.9)	148	17.53 (7.57)	72	18.66 (7.3)	−	−	−	−	−	−	2/148		
Gudur, A. R., et al. [50]	−	−	−	−	−	−	−	−	−	−	−	−	86/6053		
Hajifathalian, K., et al. [51]	114	14.6 (7.07)*	100	15.6 (8.92)*	−	−	78	15.5 (10.13)*	−	−	−	75/100	0/118	60 (74%) >10% TBWL at 2 years; improvement in ALT, AST, HSI, NAFLD fib score, HbA1c, HOMA−IR and Leptin	
Hill, C., et al. [52]	−	−	−	−	−	−	−	−	−	−	−	−	1/31	Learning curve plateau at 7 cases	
Jagtap, N., et al. [53]	26	11.33 (4.99)	26	18.07 (3.35)	−	−	−	−	−	−	−	−	0/26	23/26 (88.4%) >15%TBWL at 12 months; improvement in ALT, HSI, NAFLD fib score, FIB−4, and APRI at 12 months	
James, T. W., et al. [54]	34	16.41 (5.4)	12	23.1 (7.5)							−	12/12	2/100		
Kumar, N., et al. (2018).	Phase 2 Phase 3 NO SAMPLE	17.3 (1.7) 16 (0.8)	Phase 2 (20) Phase 3 (44)	17.3 (2.6) 17.6 (2.1)	−	−	−	−	−	−	−	−	0/122		
Li, R., et al. [56]	12	11.3 (4.7)	7	12.2 (8.9)	−	−	−	−	−	−	−	−	1/24		
Lopez−Nava, G., et al. [57]	Class I = 99 Class II = 151 Class III = 146	14.9 (6.5) 16.8 (6.3) 16.8 (6.3)	Class I = 50 Class II = 77 Class III = 84	17.1 (6.7) 19 (8) 22.2 (9.3)	−	−	−	−	−	−	−	42/50 67/77 78/84	6/435		
Lopez−Nava, G., et al. [59]	12	13.3 (7)	−	−	−	−	−	−	−	−	−	−	0/12		
Lopez−Nava, G., et al. [59]	215	15.17 (7.66)*	−	−	−	−	57	18.6 (11.15)*	−	−	−	48/57	5/248		
Manos, T., et al. [61]	84	22.4 (8**)	69	18.7 (8**)	−	−	−	−	−	−	−	−	2/191		
Matteo, M. V., et al. [61]	18	14.97 (4.56)*	12	15.27 (5.25)*	10	16 (7.38)*	10	15.55 (6.93)*	−	−	−	−	0/18		
Maydeo, A., et al. [16]	52	17.1 (4.3)	−	−	−	−	−	−	−	−	−	−	0/58		
Mehta, A., et al. [62]	47	14.8 (5.3)	39	15.5 (7.2)	47	15.4 (9.8)	−	−	−	−	−	−	0/50	Significant improvement in depression scores	
Neto, M. G., et al. [63]	178	16.9 (6.2)	123	19.7 (5.7)	−	−	−	−	−	−	−	−	0/233		
Neto, M. G., et al. [64]	−	−	−	−	−	−	−	−	−	−	−	−	15/1828		
Novikov, A. A., et al. [65]	61	14.37 (7)	28	17.57 (8.1)	−	−	−	−	−	−	−	−	1/91		
Pizzicannella, M., et al. [66]	87	13.2 (7.4)	41	13.1 (8.1)	−	−	−	−	−	−	−	−	−	Experience improved the proportion of intact ESG at 12 months	
Rapaka, B., et al. [67]	−	−	−	−	−	−	−	−	−	−	−	−	−	Delay in T50 correlated with %TBWL at 3 months	
Sarkar, A., et al. [68]	52	17.4 (6.5**)	−	−	−	−	−	−	−	−	−	−	−		
Sartoretto, A., et al. [69]	52	14.9 (6.1)	−	−	−	−	−	−	−	−	−	−	3/112		
Saumoy, M., et al. [70]	74	13.43 (7.4)	60	15.8 (9.5)	−	−	−	−	−	−	−	−	2/128	Learning curve: efficiency at 29 and mastery at 55 cases	
Sharaiha, R. Z., et al. [71]	−	−	142	15.6 (9.11)*	−	−	−	−	36 months = 68 60 months = 56	14.9 (11.77)* 15.9 (16.79)*	118/142	103/142	3/216		
***Total***	**5227 **	**15.66 (SE 0.35)**	**4118**	**17.56 (SE 0.39)**	**207**	**16.25 (SE 0.95)**	**2123**	**15.2 (SE 0.93)**	**36 months = 922**	**36 months = 14.07 (SE 0.39)**	**422/478 (88.3%)**	**632/768 (82.3%)**	**194/15,398 (1.25%)**		

* Calculated fields

** Inputted data

## Summary of Weight Loss Outcomes After ESG (Table 6 and Fig. 3)

**Table 6:** Summary of weight loss outcomes after ESG

Time	Mean %EWL	Mean %TBWL
6 months	48.04% (SE 3.59, 95%CI 40.98–55.09, 24 articles, 4329 patients)	15.66% (SE 0.35, 95%CI 14.95–16.36, 33 articles, 5227 patients)
12 months	53.09% (SE 4.15, 95%CI 44.95–61.23, 21 articles, 3652 patients)	17.56% (SE 0.39, 95%CI 16.8–18.32, 27 articles, 4118 patients)
18 months	57.98% (SE 7.38, 95%CI 43.5–72.46, 6 articles, 215 patients)	16.25% (SE 0.95, 95%CI 14.38–18.13, 5 articles, 207 patients)
24 months	46.57% (SE 9.85, 95%CI 27.26–65.88, 6 articles, 2070 patients)	15.2% (SE 0.93, 95%CI 13.36–17.04, 6 articles, 2123 patients)
36 months	53.18% (SE 7.25, 2 articles, 922 patients)	14.07% (SE 0.39, 2 articles, 922 patients)
60 months	45.3 (SD 47.32, 1 article, 56 patients)	15.9 (SD 16.79, 1 article, 56 patients)

*SE standard errors, *CI* confidence interval, *EWL* excess weight loss, *TBWL* total body weight loss

**Fig. 3:**
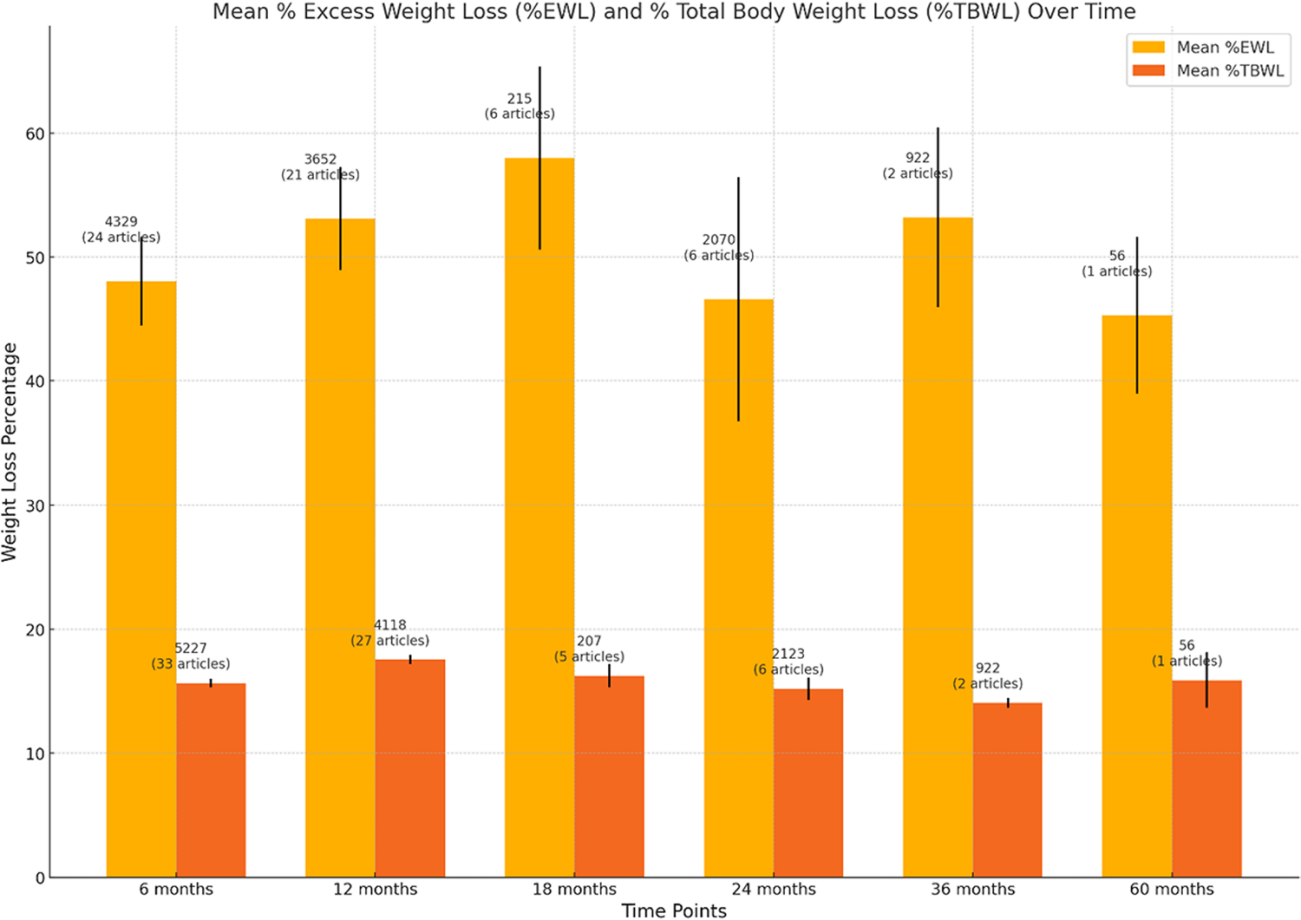
Figure displaying the mean percentage of excess weight loss (%EWL) and total body weight loss (%TBWL) over different time points following endoscopic sleeve gastroplasty (ESG) with standard error bars. The sample sizes and the number of articles at each time point are incorporated above the bars for clarity and additional context

## Quality of Evidence Assessment

All pooled outcomes were assessed for the quality of evidence according to the GRADE methodology. Since this analysis included only non-comparative data, all endpoints were rated as VERY LOW quality of evidence. Table 7 depicts the GRADE assessment.

**Table 7:**
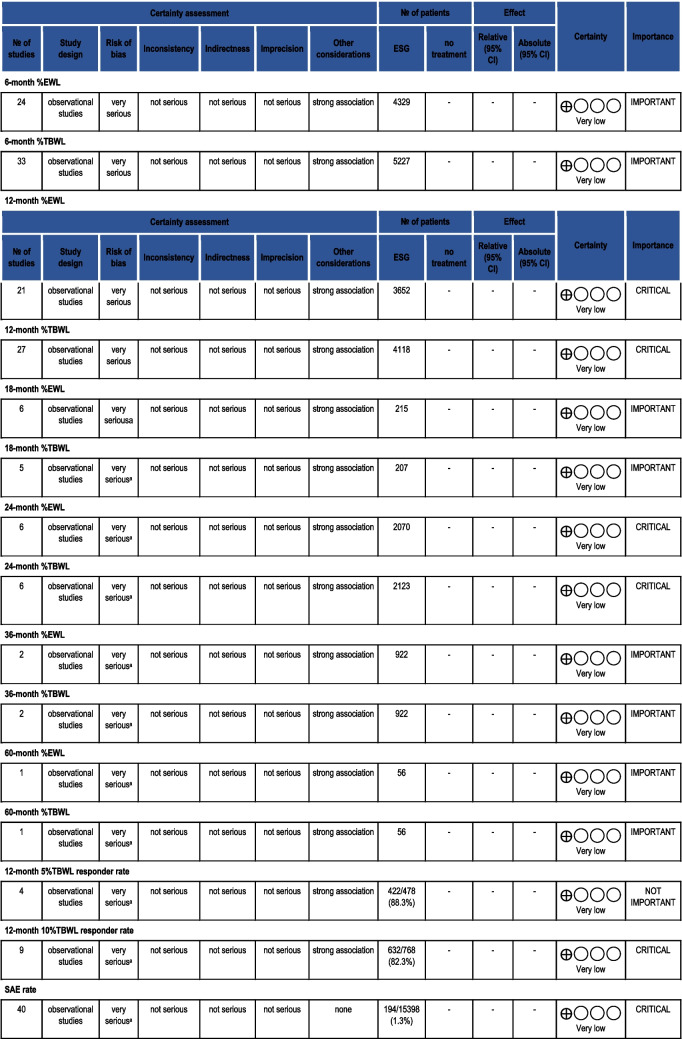
GRADE assessment of the quality of evidence for the non-comparative meta-analysis

(a) Data from case series

## Direct Comparative ESG Studies

### Systematic Review

Two independent researchers (VOB and NJ) ran separate literature searches assessing eligible studies. We searched MEDLINE (PubMed), EMBASE, and gray literature from January 1, 2013 (the year ESG was described), to October 1, 2022. The step-by-step construction of the search strategy is provided in *SUPPL 3*. The final strategy was as follows:

- MEDLINE (PubMed):(excess weight) OR (overweight) OR (obesity) AND (endoscopy) OR (endoscopic) OR (transoral*) OR (peroral*) OR (incisionless) AND (sleeve) OR (overstitch) OR (gastroplasty) OR (gastric plication) OR (gastric imbrication) AND (lifestyle) OR (diet) OR (exercise) OR (counseling) OR (sham) OR (placebo)
- EMBASE: *endoscopic AND sleeve AND gastroplasty OR (apollo AND overstitch) AND [embase]/lim NOT ([embase]/lim AND [medline]/lim) AND ('article'/it OR 'article in press'/it OR 'conference review'/it OR 'note'/it OR 'review'/it)*

The eligibility criteria included:

- Articles published online from 01/JAN/2013 until 01/OCT/2022 (last search update);
- ESG performed with the Apollo Overstitch device (no restriction as to stitching pattern);
- No language restriction;
- Full-text articles only;
- Comparative study designs: cohort studies, case–control studies, and randomized trials;
- Studies reporting efficacy and/or safety data.

**Fig. 4:**
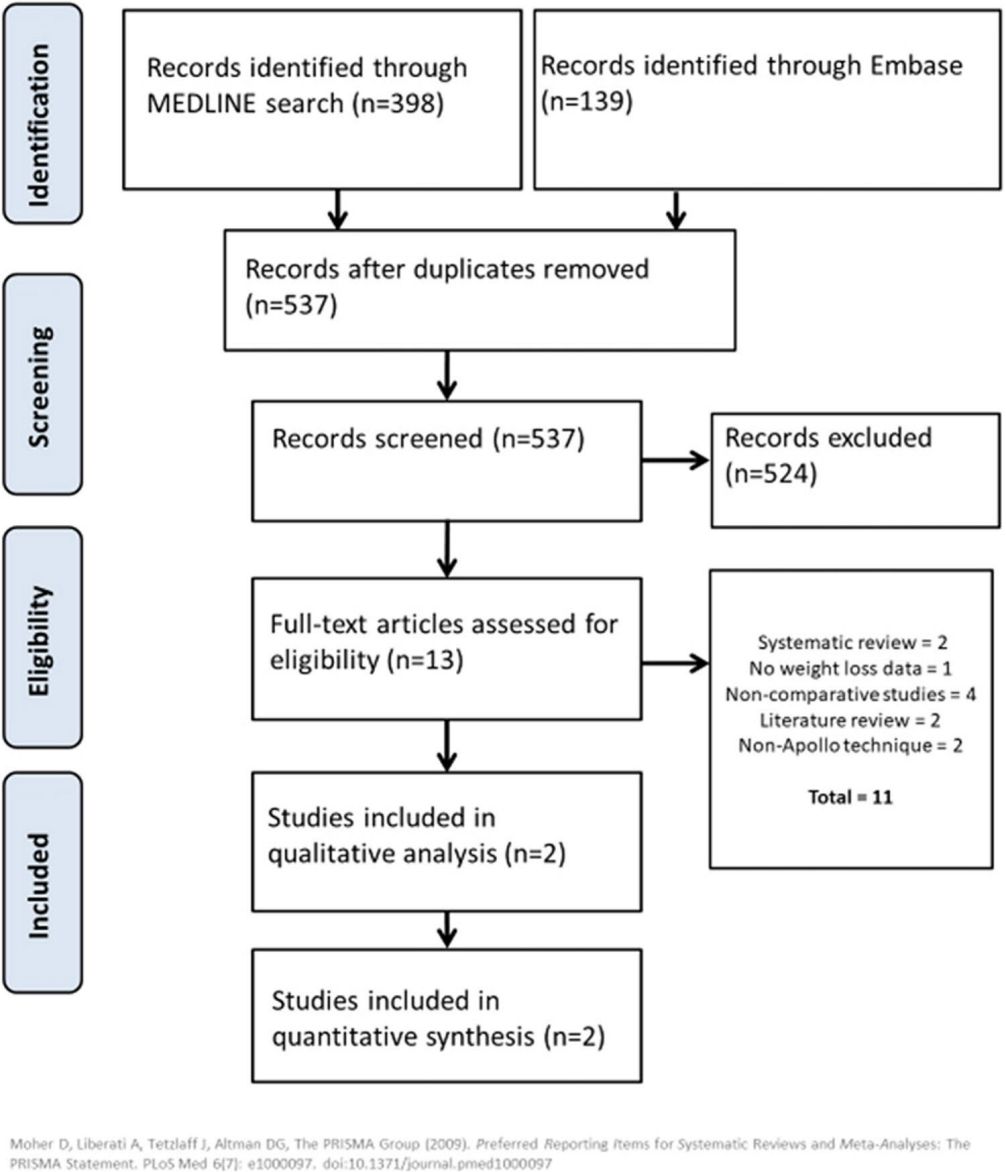
PRISMA flowchart for the literature screening and inclusion/exclusion process for only comparing ESG and lifestyle intervention

The initial search retrieved 537 records. After screening titles and abstracts, 13 articles were selected for full-text assessment. Finally, only 2 articles were included in the qualitative and quantitative analyses. Figure 4 shows the screening and inclusion/exclusion flowchart.

#### Descriptive Analysis

Cheskin et al. [40] and Abu Dayyeh et al. [22] were eligible studies for directly comparing ESG and lifestyle intervention. The first was a case-matched (1 ESG: 2–3 controls) cohort study comparing ESG plus low-intensity diet and lifestyle therapy (LIDLT) versus high-intensity diet and lifestyle therapy (HIDLT). This study included patients with obesity class 1 or higher. For both groups, patients paid out-of-pocket for the treatment (total cost ESG: U$ 16,000; total cost HIDLT: U$ 3200). A total of 386 patients (105 ESG, 281 control) were included in the study, with similar baseline characteristics. The final follow-up visit was at 12 months.

Abu Dayyeh et al. [22] was a multicenter, US FDA–regulated, open-label, randomized trial comparing ESG plus lifestyle interventions to lifestyle intervention alone (MERIT Trial). Only patients with obesity classes I and II (BMI 30–40 kg/m2) were included and allocated to ESG or control group in a 1–1.5 ratio. After 52 weeks, compliant control patients crossed over to ESG. Two hundred and nine patients (85 ESG, 124 control) were enrolled and had similar baseline characteristics. The primary endpoints were %EWL and %TBWL at 12 months, but there was an extended follow-up at 24 months for the intervention group and a 12-month follow-up for the control group crossing over to intervention.

The baseline data and the critical appraisal/risk of bias assessment for the studies are summarized in Tables 1, 3, and 4.

#### *Meta-analysis*

The two studies differ in design (cohort vs. RCT) and population (non-specified obesity vs. class I and II). According to the Cochrane Handbook [25], data from different study designs should not be pooled when few eligible studies exist. Therefore, we analyzed data from Cheskin et al. 2020 and Abu Dayyeh et al. 2022 separately. Since we could not pool data from different studies, heterogeneity, and sensitivity analyses do not apply.

**Fig. 5:**
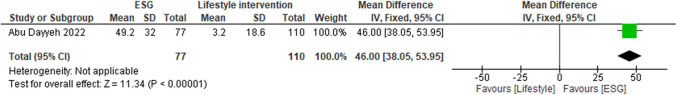
Forest plot for %EWL at 12 months in comparing ESG vs. lifestyle intervention for patients with class I and II obesity
    MD (%TBWL): 13.10 [11.08–15.12, 95%CI] – Fig. 6

A) Outcomes from MERIT Trial [22]
  At 12 months, the mean difference in weight loss outcomes compared to moderate-intensity lifestyle control was.
    MD (%EWL): 46.00 [38.05–53.95, 95%CI] – Fig. 5

**Fig. 6:**
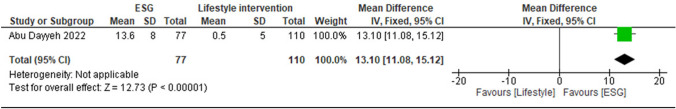
Forest plot for %TBWL at 12 months in comparing ESG vs. lifestyle intervention for patients with class I and II obesity
    SAE rate was 2% without mortality or need for intensive care or surgical intervention

The quality of evidence Abu Dayyeh et al. generated was MODERATE according to the GRADE methodology. Overall, data coming from a single study (imprecision) and the absence of double blinding were the two factors downgrading the quality of evidence. Table 8 summarizes the GRADE assessment.

**Table 8:**
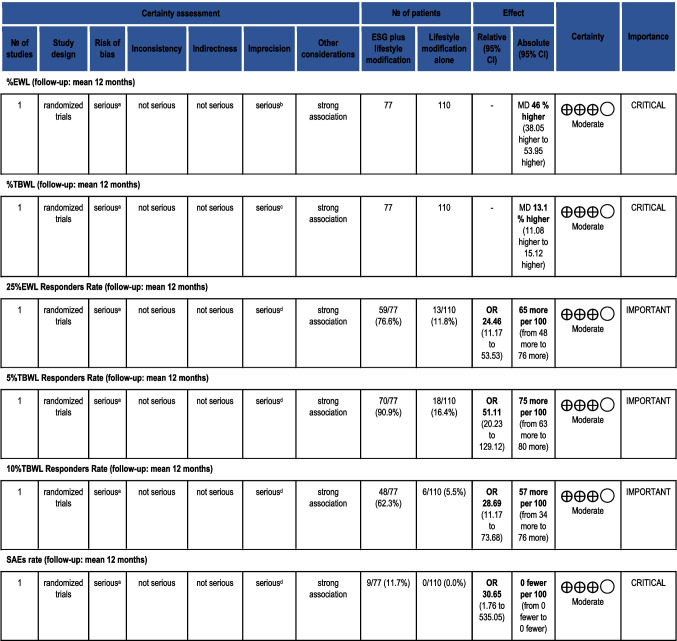
GRADE assessment  of the quality of evidence for comparing ESG vs. lifestyle intervention for patients with mild and moderate obesity

*CI* confidence interval, *MD* mean difference, *OR* odds ratio. (a) Open-label trial (detection bias) and loss to follow-up rates (20% intervention, 29% control group). Attrition bias present. (b) Single RCT with 77 patients in the intervention arm and 110 in the control arm. Large SDs: mean 49.2 ± 32 versus 3.2 ± 18.6. (c) Single RCT with 77 patients in the intervention arm and 110 in the control arm. Large SD: mean 13.6 ± 8 versus 0.8 ± 5. (d) Single RCT with 77 patients in the intervention arm and 110 in the control arm

**Fig. 7:**
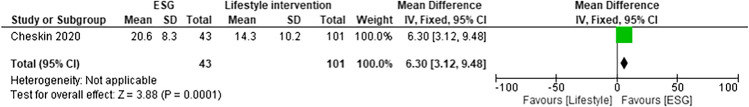
Forest plot for %TBWL at 12 months in the comparison of ESG vs. high-intensity lifestyle intervention for patients with obesity (all classes)
    Adverse events rate in the ESG group was 4.8%, with no mortality, need for intensive care, or surgical intervention.

B) Outcomes from Cheskin et al. (ADD REF here)
  At 12 months, the mean difference in weight loss outcomes compared to high-intensity lifestyle control [40].
    MD (%TBWL): 6.3 [3.12-9.48, 95%CI] – Figure 7

The quality of evidence Cheskin et al. generated was VERY LOW according to the GRADE methodology. Overall, data from a single study (imprecision) and a non-randomized study design (selection bias) led to the final quality of evidence. Table 9 summarizes the GRADE assessment.

**Table 9:**
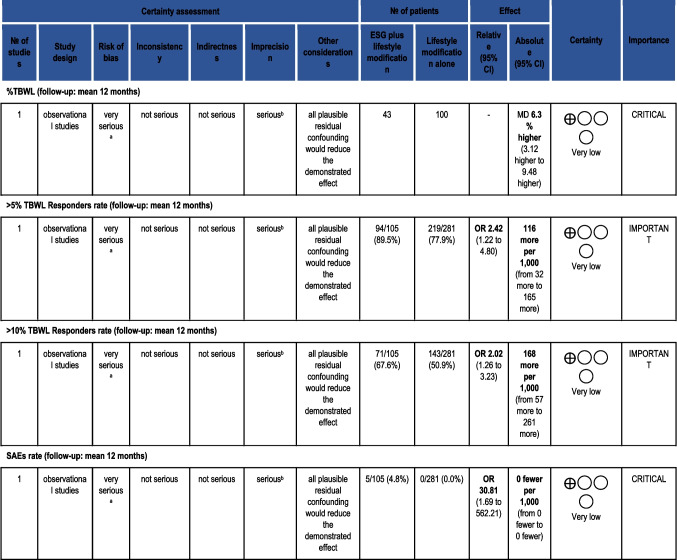
GRADE assessment of the quality of evidence for comparing ESG vs. lifestyle intervention for patients with mild and moderate obesity. Question: ESG plus lifestyle modification compared to Lifestyle modification alone for obesity

*CI* confidence interval, *MD* mean difference, *OR* odds ratio. (a) Selection bias (matched controls). Channeling bias. Confounding variables (socio-economic status), high loss to follow-up rates. (b) Single study, small total sample size/small number of events, large SDs

## IFSO Bariatric Endoscopy Position Statement and Future Direction

Based on a comprehensive systematic review and meta-analysis, the IFSO Bariatric Endoscopy Committee endorses endoscopic sleeve gastroplasty (ESG) as an effective and valuable intervention for managing obesity. ESG is particularly beneficial for patients with class I and II obesity, as well as for those with class III obesity who are not suitable candidates for traditional MBS. This minimally invasive procedure not only achieves significant weight loss outcomes in the short and mid-terms but also maintains a favorable safety profile, as evidenced by a low incidence of serious adverse events.

The systematic review encompassed numerous observational studies, which, despite being categorized as very low quality, consistently reported positive and similar outcomes from different cohorts and practice settings globally, indicating reproducibility, generalizability, and maturity of ESG. Additionally, including a single randomized controlled trial in the meta-analysis provided moderate quality evidence, further substantiating the efficacy and safety of ESG. This dual-source evidence base enhances the robustness of the findings, lending more significant support to the recently published IFSO International Delphi Consensus statement on the position of ESG in the spectrum of obesity care [23].

It is important to emphasize the crucial role of integrating and complementing any obesity intervention, such as ESG, with a comprehensive and longitudinal healthy living program. This program should include a healthy diet, physical activity, adequate sleep, and mindfulness to maintain the weight loss benefits and maximize the overall impact on health by the intervention. By incorporating ESG into a comprehensive program, healthcare providers can offer a broader spectrum of options for obesity management tailored to the needs and circumstances of individual patients. This integrated approach enhances the effectiveness of ESG but also ensures long-term health benefits for patients.

## Future Direction

In this report, we investigated endoscopic sleeve gastroplasty utilizing the Apollo Overstitch™ platform (Boston Scientific, Marlborough, MA, USA) based on the maturity of the technique, regulatory approvals (https://www.accessdata.fda.gov/cdrh_docs/pdf21/DEN210045.pdf, https://www.nice.org.uk/guidance/ipg783) and cost-effectiveness [72–74]. Other endoscopic gastric remodeling techniques, including the Primary Obesity Surgery Endoluminal 2.0 (USGI Medical, San Clemente, CA), Endomina™ Gastric Plication (Endo Tools, Gosselies, Belgium), and the Endozip™ automated suturing device (Caesarea, Israel), are at different stages of clinical trials and evidence generation and are demonstrating similar safety and efficacy profiles. The committee will update its position statement to reflect and incorporate the evolving evidence base as the clinical evidence continues to mature for these procedures.

Advancements in obesity management medication now offer effective options for selected patients. The value proposition and comparative effectiveness of ESG compared to, or in addition to, obesity pharmacotherapies is an active area of investigation. Observational studies have demonstrated the benefits of combining or sequencing ESG with obesity pharmacotherapies, particularly in enhancing the durability of the response [75].

However, given the limited follow-up in the existing literature—typically extending to five years or less—additional data is required to better understand the different archetypes of response to ESG over the long term. This data will also help in defining optimal personalized approaches to maximize the durability of the response and improve long-term health outcomes.

## Data Availability

No datasets were generated or analysed during the current study.

## References

[1] Worldwide trends in body-mass index. underweight, overweight, and obesity from 1975 to 2016: a pooled analysis of 2416 population-based measurement studies in 128·9 million children, adolescents, and adults. Lancet. 2017;390:2627–42. 29029897 10.1016/S0140-6736(17)32129-3 PMC5735219

[2] World Healh Organization. Overweight and obesity - global observatory data. https://www.who.int/news-room/fact-sheets/detail/obesity-and-overweight.

[3] Hales CM, Carroll MD, Fryar CD, et al. Prevalence of obesity among adults and youth: United States, 2015–2016. NCHS Data Brief. 2017;288:1–8. 29155689

[4] Hales CM, Fryar CD, Carroll MD, et al. Trends in obesity and severe obesity prevalence in US youth and adults by sex and age, 2007–2008 to 2015–2016. JAMA. 2018;319:1723–5. 29570750 10.1001/jama.2018.3060 PMC5876828

[5] Finkelstein EA, Khavjou OA, Thompson H, et al. Obesity and severe obesity forecasts through 2030. Am J Prev Med. 2012;42:563–70. 22608371 10.1016/j.amepre.2011.10.026

[6] Eisenberg D, Shikora SA, Aarts E, et al. American Society for Metabolic and Bariatric Surgery (ASMBS) and International Federation for the Surgery of Obesity and Metabolic Disorders (IFSO): indications for metabolic and bariatric surgery. Surg Obes Relat Dis. 2022;18:1345–56. 36280539 10.1016/j.soard.2022.08.013

[7] Carlsson LMS, Sjöholm K, Jacobson P, et al. Life expectancy after bariatric surgery in the Swedish obese subjects study. N Engl J Med. 2020;383:1535–43. 33053284 10.1056/NEJMoa2002449 PMC7580786

[8] Angrisani L, Santonicola A, Iovino P, et al. Bariatric Surgery Survey 2018: similarities and disparities among the 5 IFSO chapters. Obes Surg. 2021;31:1937–48. 33432483 10.1007/s11695-020-05207-7 PMC7800839

[9] Angrisani L, Santonicola A, Iovino P, et al. IFSO Worldwide Survey 2016: primary, endoluminal, and revisional procedures. Obes Surg. 2018;28:3783–94. 30121858 10.1007/s11695-018-3450-2

[10] Ogden CL, Carroll MD, Kit BK, et al. Prevalence of childhood and adult obesity in the United States, 2011–2012. JAMA. 2014;311:806–14. 24570244 10.1001/jama.2014.732 PMC4770258

[11] Whitlock G, Lewington S, Sherliker P, et al. Body-mass index and cause-specific mortality in 900 000 adults: collaborative analyses of 57 prospective studies. Lancet (London, England). 2009;373:1083–96. 19299006 10.1016/S0140-6736(09)60318-4 PMC2662372

[12] Abu Dayyeh BK, Rajan E, Gostout CJ. Endoscopic sleeve gastroplasty: a potential endoscopic alternative to surgical sleeve gastrectomy for treatment of obesity. Gastrointest Endosc. 2013;78:530–5. 23711556 10.1016/j.gie.2013.04.197

[13] Galvao-Neto MDP, Grecco E, Souza TFd, et al. Endoscopic sleeve gastroplasty - minimally invasive therapy for primary obesity treatment. Arquivos brasileiros de cirurgia digestiva : ABCD = Brazilian Archiv of Digestive Surgery. 2016;29Suppl 1:95–97. 10.1590/0102-6720201600S10023 PMC5064280 27683786

[14] Lopez-Nava G, Galvão MP, Bautista-Castaño I, et al. Endoscopic sleeve gastroplasty: how i do it? Obes Surg. 2015;25:1534–8. 26003549 10.1007/s11695-015-1714-7

[15] Vargas EJ, Rizk M, Gomez-Villa J, et al. Effect of endoscopic sleeve gastroplasty on gastric emptying, motility and hormones: a comparative prospective study. Gut 2022. 10.1136/gutjnl-2022-327816 10.1136/gutjnl-2022-327816 PMC10102256 36241388

[16] Maydeo A, Patil G, Dalal A, et al. An Indian experience of endoscopic treatment of obesity by using a novel technique of endoscopic sleeve gastroplasty (accordion procedure). J Assoc Physicians India. 2020;68:14–7. 32738834

[17] Graus Morales J, Crespo Perez L, Marques A, et al. Modified endoscopic gastroplasty for the treatment of obesity. Surg Endosc. 2018;32:3936–42. 29492709 10.1007/s00464-018-6133-0

[18] Espinet-Coll E, Nebreda-Durán J, Galvao-Neto M, et al. Suture pattern does not influence outcomes of endoscopic sleeve gastroplasty in obese patients. Endoscopy international open. 2020;8:E1349–58. 33015337 10.1055/a-1221-9835 PMC7508658

[19] López-Nava Breviere G, Bautista-Castaño I, Fernández-Corbelle JP, et al. Endoscopic sleeve gastroplasty (the Apollo method): a new approach to obesity management. Rev Esp Enferm Dig. 2016;108:201–6. 26900986 10.17235/reed.2016.3988/2015

[20] Alqahtani A, Al-Darwish A, Mahmoud AE, et al. Short-term outcomes of endoscopic sleeve gastroplasty in 1000 consecutive patients. Gastrointest Endosc. 2019;89:1132–8. 30578757 10.1016/j.gie.2018.12.012

[21] Cheskin LJ, Hill C, Adam A, et al. Endoscopic sleeve gastroplasty versus high-intensity diet and lifestyle therapy: a case-matched study. Gastrointest Endosc. 2020;91:342-349.e1. 31568769 10.1016/j.gie.2019.09.029

[22] Abu Dayyeh BK, Bazerbachi F, Vargas EJ, et al. Endoscopic sleeve gastroplasty for treatment of class 1 and 2 obesity (MERIT): a prospective, multicentre, randomised trial. Lancet. 2022;400:441–51. 35908555 10.1016/S0140-6736(22)01280-6

[23] Salminen P, Kow L, Aminian A, et al. IFSO consensus on definitions and clinical practice guidelines for obesity management-an international delphi study. Obes Surg. 2024;34:30–42. 37999891 10.1007/s11695-023-06913-8 PMC10781804

[24] Moher D, Liberati A, Tetzlaff J, et al. Preferred reporting items for systematic reviews and meta-analyses: the PRISMA statement. BMJ (Clinical research ed). 2009;339:b2535–b2535. 19622551 10.1136/bmj.b2535 PMC2714657

[25] Higgins JPT, Thomas J, Chandler J, Cumpston M, Li T, Page MJ, Welch VA. Cochrane Handbook for Systematic Reviews of Interventions (Version 6.3, updated February 2022). Cochrane. 2022. Available from: https://training.cochrane.org/handbook.

[26] The Joanna Briggs Institute. Joanna Briggs Institute Reviewers' Manual: 2016 ed. Australia: the joanna briggs institute. 2016. Available from: https://jbi.global/research/handbook.

[27] Wells GA, Shea B, O'Connell D, et al. The Newcastle-Ottawa Scale (NOS) for assessing the quality of nonrandomised studies in meta-analyses [Internet]. 2021. Available from: http://www.ohri.ca/programs/clinical_epidemiology/oxford.asp.

[28] Jadad AR, Moore RA, Carroll D, et al. Assessing the quality of reports of randomized clinical trials: is blinding necessary? Control Clin Trials. 1996;17:1–12. 8721797 10.1016/0197-2456(95)00134-4

[29] Hozo SP, Djulbegovic B, Hozo I. Estimating the mean and variance from the median, range, and the size of a sample. BMC Med Res Methodol. 2005;5:13–13. 15840177 10.1186/1471-2288-5-13 PMC1097734

[30] Schünemann H, Brożek J, Guyatt G, Oxman A. GRADE handbook for grading quality of evidence and strength of recommendations. The GRADE Working Group. 2013. Available from: https://gdt.gradepro.org/app/handbook/handbook.html.

[31] Abu Dayyeh BK, Acosta A, Camilleri M, et al. Endoscopic sleeve gastroplasty alters gastric physiology and induces loss of body weight in obese individuals. Clin Gastroenterol Hepatol. 2017;15:37-43.e1. 26748219 10.1016/j.cgh.2015.12.030

[32] Alqahtani A, Elahmedi M, Alqahtani YA, et al. Endoscopic sleeve gastroplasty in 109 consecutive children and adolescents with obesity: two-year outcomes of a new modality. Am J Gastroenterol. 2019;114:1857–62. 31658128 10.14309/ajg.0000000000000440

[33] Alqahtani AR, Elahmedi M, Aldarwish A, et al. Endoscopic gastroplasty versus laparoscopic sleeve gastrectomy: a noninferiority propensity score-matched comparative study. Gastrointest Endosc. 2022;96:44–50. 35248571 10.1016/j.gie.2022.02.050

[34] Asokkumar R, Lim CH, Tan AS, et al. Safety and early efficacy of endoscopic sleeve gastroplasty (ESG) for obesity in a multi-ethnic Asian population in Singapore. JGH Open. 2021;5:1351–6. 34950778 10.1002/jgh3.12680 PMC8674547

[35] Badurdeen D, Hoff AC, Hedjoudje A, et al. Endoscopic sleeve gastroplasty plus liraglutide versus endoscopic sleeve gastroplasty alone for weight loss. Gastrointest Endosc. 2021;93:1316-1324.e1. 33075366 10.1016/j.gie.2020.10.016

[36] Barrichello S, Hourneaux de Moura DT, Hourneaux de Moura EG, et al. Endoscopic sleeve gastroplasty in the management of overweight and obesity: an international multicenter study. Gastrointest Endosc 2019;90:770–780. 10.1016/j.gie.2019.06.013 31228432

[37] Bhandari M, Jain S, Mathur W, et al. Endoscopic sleeve gastroplasty is an effective and safe minimally invasive approach for treatment of obesity: first Indian experience. Digestive endoscopy : official journal of the Japan Gastroenterological Endoscopy Society 2019. 10.1111/den.13508 10.1111/den.13508 31394006

[38] Callahan ZM, Su B, Kuchta K, et al. Endoscopic suturing results in high technical and clinical success rates for a variety of gastrointestinal pathologies. J Gastrointest Surg. 2020;24:278–87. 31823323 10.1007/s11605-019-04485-6

[39] Carr P, Keighley T, Petocz P, et al. Efficacy and safety of endoscopic sleeve gastroplasty and laparoscopic sleeve gastrectomy with 12+ months of adjuvant multidisciplinary support. BMC Prim Care. 2022;23:26. 35123409 10.1186/s12875-022-01629-7 PMC8817771

[40] Cheskin LJ, Hill C, Adam A, et al. Endoscopic sleeve gastroplasty versus high-intensity diet and lifestyle therapy: a case-matched study. Gastrointest Endosc. 2020;91:342-349.e1. 31568769 10.1016/j.gie.2019.09.029

[41] Espinet-Coll E, Díaz-Galán P, Nebreda-Durán J, et al. Persistence of sutures and gastric reduction after endoscopic sleeve gastroplasty: radiological and endoscopic assessment. Obes Surg. 2022;32:1969–79. 35353330 10.1007/s11695-022-06039-3

[42] Espinet-Coll E, Nebreda-Durán J, Galvao-Neto M, et al. Suture pattern does not influence outcomes of endoscopic sleeve gastroplasty in obese patients. Endosc Int Open. 2020;8:E1349-e1358. 33015337 10.1055/a-1221-9835 PMC7508658

[43] Farha J, McGowan C, Hedjoudje A, et al. Endoscopic sleeve gastroplasty: suturing the gastric fundus does not confer benefit. Endoscopy. 2021;53:727–31. 32777827 10.1055/a-1236-9347

[44] Fayad L, Adam A, Schweitzer M, et al. Endoscopic sleeve gastroplasty versus laparoscopic sleeve gastrectomy: a case-matched study. Gastrointest Endosc. 2019;89:782–8. 30148991 10.1016/j.gie.2018.08.030

[45] Fayad L, Cheskin LJ, Adam A, et al. Endoscopic sleeve gastroplasty versus intragastric balloon insertion: efficacy, durability, and safety. Endoscopy 2019. 10.1055/a-0852-3441 10.1055/a-0852-3441 30841009

[46] Fiorillo C, Quero G, Vix M, et al. 6-month gastrointestinal quality of life (QoL) results after endoscopic sleeve gastroplasty and laparoscopic sleeve gastrectomy: a propensity score analysis. Obes Surg. 2020;30:1944–51. 31965488 10.1007/s11695-020-04419-1

[47] Ghoz H, Bryant M, Fritz H, et al. Endoscopic sleeve gastroplasty and postprocedural nutritional deficiencies: results from a single center exploratory study. Eur J Gastroenterol Hepatol. 2021;33:e1039–41. 35048661 10.1097/MEG.0000000000002316

[48] Glaysher MA, Moekotte AL, Kelly J. Endoscopic sleeve gastroplasty: a modified technique with greater curvature compression sutures. Endosc Int Open. 2019;7:E1303-e1309. 31595224 10.1055/a-0996-8089 PMC6779570

[49] Graus Morales J, Crespo Pérez L, Marques A, et al. Modified endoscopic gastroplasty for the treatment of obesity. Surg Endosc. 2018;32:3936–42. 29492709 10.1007/s00464-018-6133-0

[50] Gudur AR, Geng C, Kshatri S, et al. Comparison of endoscopic sleeve gastroplasty versus surgical sleeve gastrectomy: a metabolic and bariatric surgery accreditation and quality improvement program database analysis. Gastrointest Endosc 2022. 10.1016/j.gie.2022.07.017 10.1016/j.gie.2022.07.017 35870507

[51] Hajifathalian K, Mehta A, Ang B, et al. Improvement in insulin resistance and estimated hepatic steatosis and fibrosis after endoscopic sleeve gastroplasty. Gastrointest Endosc. 2021;93:1110–8. 32861753 10.1016/j.gie.2020.08.023

[52] Hill C, El Zein M, Agnihotri A, et al. Endoscopic sleeve gastroplasty: the learning curve. Endosc Int Open. 2017;5:E900-e904. 28924597 10.1055/s-0043-115387 PMC5597932

[53] Jagtap N, Kalapala R, Katakwar A, et al. Endoscopic sleeve gastroplasty - minimally invasive treatment for non-alcoholic fatty liver disease and obesity. Indian J Gastroenterol. 2021;40:572–9. 34914039 10.1007/s12664-021-01202-7

[54] James TW, Reddy S, Vulpis T, et al. Endoscopic sleeve gastroplasty is feasible, safe, and effective in a non-academic setting: short-term outcomes from a community gastroenterology practice. Obes Surg. 2020;30:1404–9. 31853865 10.1007/s11695-019-04331-3

[55] Kumar N, Abu Dayyeh BK, Lopez-Nava Breviere G, et al. Endoscopic sutured gastroplasty: procedure evolution from first-in-man cases through current technique. Surg Endosc. 2018;32:2159–64. 29075966 10.1007/s00464-017-5869-2 PMC5845469

[56] Li R, Veltzke-Schlieker W, Adler A, et al. Endoscopic sleeve gastroplasty (ESG) for high-risk patients, high body mass index (> 50 kg/m(2)) patients, and contraindication to abdominal surgery. Obes Surg. 2021;31:3400–9. 33905069 10.1007/s11695-021-05446-2

[57] Lopez-Nava G, Laster J, Negi A, et al. Endoscopic sleeve gastroplasty (ESG) for morbid obesity: how effective is it? Surg Endosc. 2022;36:352–60. 33492503 10.1007/s00464-021-08289-1

[58] Lopez-Nava G, Negi A, Bautista-Castaño I, et al. Gut and metabolic hormones changes after endoscopic sleeve gastroplasty (ESG) vs. laparoscopic sleeve gastrectomy (LSG). Obes Surg. 2020;30:2642–51. 32193741 10.1007/s11695-020-04541-0

[59] Lopez-Nava G, Sharaiha RZ, Vargas EJ, et al. Endoscopic sleeve gastroplasty for obesity: a multicenter study of 248 patients with 24 months follow-up. Obes Surg. 2017;27:2649–55. 28451929 10.1007/s11695-017-2693-7

[60] Manos T, Costil V, Karsenty L, et al. Safety of endoscopic sleeve gastroplasty with a single-channel endoscope. Obes Surg. 2022;32:3074–8. 35857182 10.1007/s11695-022-06210-w

[61] Matteo MV, Bove V, Pontecorvi V, et al. Outcomes of endoscopic sleeve gastroplasty in the elder population. Obes Surg 2022. 10.1007/s11695-022-06232-4 10.1007/s11695-022-06232-4 PMC9532333 35918595

[62] Mehta A, Hajifathalian K, Shah SL, et al. Quality of life, mental health, and weight loss outcomes following endoscopic sleeve gastroplasty. J Gastrointestinal Surg : Off J Soc Surg Alimentary Tract. 2022;26:469–71. 10.1007/s11605-021-05137-4 34506034

[63] Neto MG, Moon RC, de Quadros LG, et al. Safety and short-term effectiveness of endoscopic sleeve gastroplasty using overstitch: preliminary report from a multicenter study. Surg Endosc. 2020;34:4388–94. 31624939 10.1007/s00464-019-07212-z

[64] Neto MG, Silva LB, de Quadros LG, et al. Brazilian consensus on endoscopic sleeve gastroplasty. Obes Surg. 2021;31:70–8. 32815105 10.1007/s11695-020-04915-4

[65] Novikov AA, Afaneh C, Saumoy M, et al. Endoscopic sleeve gastroplasty, laparoscopic sleeve gastrectomy, and laparoscopic band for weight loss: how do they compare? J Gastrointest Surg. 2018;22:267–73. 29110192 10.1007/s11605-017-3615-7

[66] Pizzicannella M, Lapergola A, Fiorillo C, et al. Does endoscopic sleeve gastroplasty stand the test of time? Objective assessment of endoscopic ESG appearance and its relation to weight loss in a large group of consecutive patients. Surg Endosc. 2020;34:3696–705. 31932925 10.1007/s00464-019-07329-1

[67] Rapaka B, Maselli DB, Lopez-Nava G, et al. Effects on physiologic measures of appetite from intragastric balloon and endoscopic sleeve gastroplasty: results of a prospective study. Chin Med J (Engl). 2022;135:1234–41. 35788090 10.1097/CM9.0000000000002097 PMC9337251

[68] Sarkar A, Tawadros A, Andalib I, et al. Safety and efficacy of endoscopic sleeve gastroplasty for obesity management in new bariatric endoscopy programs: a multicenter international study. Ther Adv Gastrointest Endosc. 2022;15:26317745221093884. 35694412 10.1177/26317745221093883 PMC9178997

[69] Sartoretto A, Sui Z, Hill C, et al. Endoscopic sleeve gastroplasty (ESG) is a reproducible and effective endoscopic bariatric therapy suitable for widespread clinical adoption: a large, international multicenter study. Obes Surg. 2018;28:1812–21. 29450845 10.1007/s11695-018-3135-x

[70] Saumoy M, Schneider Y, Zhou XK, et al. A single-operator learning curve analysis for the endoscopic sleeve gastroplasty. Gastrointest Endosc. 2018;87:442–7. 28843586 10.1016/j.gie.2017.08.014

[71] Sharaiha RZ, Hajifathalian K, Kumar R, et al. Five-year outcomes of endoscopic sleeve gastroplasty for the treatment of obesity. Clin Gastroenterol Hepatol. 2021;19:1051-1057.e2. 33011292 10.1016/j.cgh.2020.09.055

[72] Kelly J, Menon V, O’Neill F, et al. UK cost-effectiveness analysis of endoscopic sleeve gastroplasty versus lifestyle modification alone for adults with class II obesity. Int J Obes (Lond). 2023;47:1161–70. 37674032 10.1038/s41366-023-01374-6 PMC10599990

[73] Saumoy M, Gandhi D, Buller S, et al. Cost-effectiveness of endoscopic, surgical and pharmacological obesity therapies: a microsimulation and threshold analyses. Gut. 2023;72:2250–9. 37524445 10.1136/gutjnl-2023-330437

[74] Haseeb M, Chhatwal J, Xiao J, et al. Semaglutide vs endoscopic sleeve gastroplasty for weight loss. JAMA Netw Open. 2024;7:e246221. 38607627 10.1001/jamanetworkopen.2024.6221 PMC11015347

[75] Gala K, Ghusn W, Brunaldi V, et al. Outcomes of concomitant antiobesity medication use with endoscopic sleeve gastroplasty in clinical US settings. Obes Pillars. 2024;11:100112. 38831924 10.1016/j.obpill.2024.100112 PMC11145356

